# A Putative Small Solute Transporter Is Responsible for the Secretion of G377 and TRAP-Containing Secretory Vesicles during *Plasmodium* Gamete Egress and Sporozoite Motility

**DOI:** 10.1371/journal.ppat.1005734

**Published:** 2016-07-18

**Authors:** Jessica Kehrer, Mirko Singer, Leandro Lemgruber, Patricia A. G. C. Silva, Friedrich Frischknecht, Gunnar R. Mair

**Affiliations:** 1 Integrative Parasitology, Center for Infectious Diseases, University of Heidelberg Medical School, Heidelberg, Germany; 2 Instituto Medicina Molecular, Lisbon, Portugal; Wellcome Trust Sanger Institute, UNITED KINGDOM

## Abstract

Regulated protein secretion is required for malaria parasite life cycle progression and transmission between the mammalian host and mosquito vector. During transmission from the host to the vector, exocytosis of highly specialised secretory vesicles, such as osmiophilic bodies, is key to the dissolution of the red blood cell and parasitophorous vacuole membranes enabling gamete egress. The positioning of adhesins from the TRAP family, from micronemes to the sporozoite surface, is essential for gliding motility of the parasite and transmission from mosquito to mammalian host. Here we identify a conserved role for the putative pantothenate transporter PAT in *Plasmodium berghei* in vesicle fusion of two distinct classes of vesicles in gametocytes and sporozoites. PAT is a membrane component of osmiophilic bodies in gametocytes and micronemes in sporozoites. Despite normal formation and trafficking of osmiophilic bodies to the cell surface upon activation, PAT-deficient gametes fail to discharge their contents, remain intraerythrocytic and unavailable for fertilisation and further development in the mosquito. Sporozoites lacking PAT fail to secrete TRAP, are immotile and thus unable to infect the subsequent rodent host. Thus, *P*. *berghei* PAT appears to regulate exocytosis in two distinct populations of vesicles in two different life cycle forms rather than acting as pantothenic transporter during parasite transmission.

## Author Summary

Transmission of the malaria parasite between mosquito and host requires two different life cycle stages—the gametocyte and the sporozoite. In both parasite forms, transmission is dependent on exocytosis of stage-specific vesicles. In gametocytes these vesicles release proteins allowing egress from red blood cells and fertilization, and are hence needed to establish an infection in the mosquito. In contrast, proteins are secreted into the membrane of the sporozoite, where they play distinct roles during adhesion and motility, both crucial for transmission back into the mammalian host. Here we show that parasites lacking the putative small solute transporter PAT are still able to form vesicles in both parasite forms but are unable to fuse and secrete their contents. This results in impaired parasite transmission into and from the mosquito. Our work shows that a single protein can regulate the function of functionally distinct classes of vesicles in different life cycle forms of a parasite.

## Introduction

Malaria is a vector-borne disease caused by parasitic protozoans from the genus *Plasmodium*. Although infection and mortality rates are decreasing, 2015 still showed more than 200 million infections and over 400,000 deaths, often children (World Malaria Report 2015, WHO). With approximately 5000 protein coding genes, this unicellular parasite, haploid throughout most of its life cycle, develops an amazing morphological variety of life cycle forms that allow invasion and infection of different hosts and cell types. The parasite is transmitted by female *Anopheles* mosquitoes and requires the formation of two specialised, but fundamentally different parasite forms: the gametocyte and sporozoite. During the blood meal the infected mosquito injects highly motile salivary gland sporozoites into a naïve host, or is itself infected if feeding on an individual harbouring gametocytes, sexual precursor cells that are intraerythrocytic [red blood cell (RBC) resident], immotile forms within the mammalian host. They are taken up into the mosquito midgut during a blood meal where they are activated to differentiate into mature gametes. The formation of free, mature gametes requires egress from the host erythrocyte in order to take part in fertilisation and the formation of zygotes that eventually produce motile ookinetes that escape the blood meal and infect the mosquito vector [1, 2]. Gamete egress requires the dissolution of two membranes: the parasitophorous vacuole (PV) membrane (PVM), which separates the RBC cytoplasm from the PV, and the host’s RBC membrane (RBCM) [3, 4]. In contrast to asexually replicating forms (merozoites), which inhabit a similar intraerythrocytic environment in the RBC and also egress periodically [5], egress of gametes occurs exclusively in the mosquito vector. Gamete egress is triggered by the change in environmental conditions from those encountered in the circulatory system of the mammalian host [4, 6, 7] and is accomplished within 15 minutes. It depends on the secretion of intracellular, vesicle-resident protein factors that facilitate lysis of the PVM and the RBCM [3, 4]. PVM dissolution requires the exocytosis of so-called osmiophilic bodies (OB), electron dense organelles derived from Golgi vesicles [8] that are known to contain three types of unrelated proteins: G377, GEST and MDV1/PEG3 [9–14]. The perforin-like protein PPLP2 has been shown to mediate lysis of the RBCM and is proposed to inhabit distinct egress vesicles [15, 16]. All four factors are secreted within minutes of transmission to the mosquito vector or *in vitro* in a medium that mimics mosquito midgut environmental conditions. G377 is expressed only in females; GEST, MDV1/PEG3 and PPLP2 are present in both sexes. Each individual protein is important, but not *per se* essential for gamete release; null mutants typically suffer reduced mosquito infection rates while transmission still occurs [9–12, 14]. Most likely these, and perhaps additional but unknown factors, are released in concert to enable rapid breakdown of the PVM and RBCM.

Sporozoites on the other hand are extracellular forms that move at high speed without changing shape. Motility requires the secretion of micronemal proteins such as TRAP (Thrombospondin Related Anonymous Protein) that provide the link between the intracellular acto-myosin motor and the extracellular substrate. Secretion and positioning of TRAP on the cell surface is essential for migration of mature sporozoites from the mosquito hemocoel into the salivary gland lumen, for migration in the skin following the mosquito bite and liver invasion. Sporozoites are a key model to understanding this unique type of motility, which can be studied *in vitro* or *in vivo*. The mechanisms underlying gliding motility are not completely understood [17–21]. However various TRAP family proteins (S6, TLP, TRAP) appear to play distinct roles during sporozoite adhesion and motility [22–25]. TRAP provides attachment to the substrate and during productive motility is cleaved by rhomboid proteases severing the link established between the parasite and the substrate, producing a regular turnover of adhesion sites [26, 27]. Parasites lacking TRAP fail to attach and remain trapped in the hemolymph unable to invade the salivary glands thus producing a block in parasite transmission [22] while mutants defective in TRAP cleavage show reduced motility [26]. TRAP-family adhesins are secreted in a calcium-dependent manner from small secretory organelles known as micronemes, which are most likely formed directly from the Golgi and accumulate at the apical part of the sporozoite [28, 29].

Here we identify a member of the major facilitator superfamily (MFS)—transporters of solutes such as sugars, protons and small metabolites [30]—as an essential component of the *Plasmodium berghei* secretion machinery. The protein PBANKA_0303900—ortholog of the *P*. *falciparum* vitamin B_5_ transporter PAT—localises to vesicles of gametocytes (osmiophilic bodies) and sporozoites (micronemes). Redundant for secretory vesicle formation, PAT is essential for the secretion of osmiophilic bodies in gametocytes and micronemes in sporozoites. Hence, the protein is key to the transmission of the malaria parasite to and from the mosquito vector.

## Results

### Expression profiling and epitope tagging of PAT

**Fig 1:**
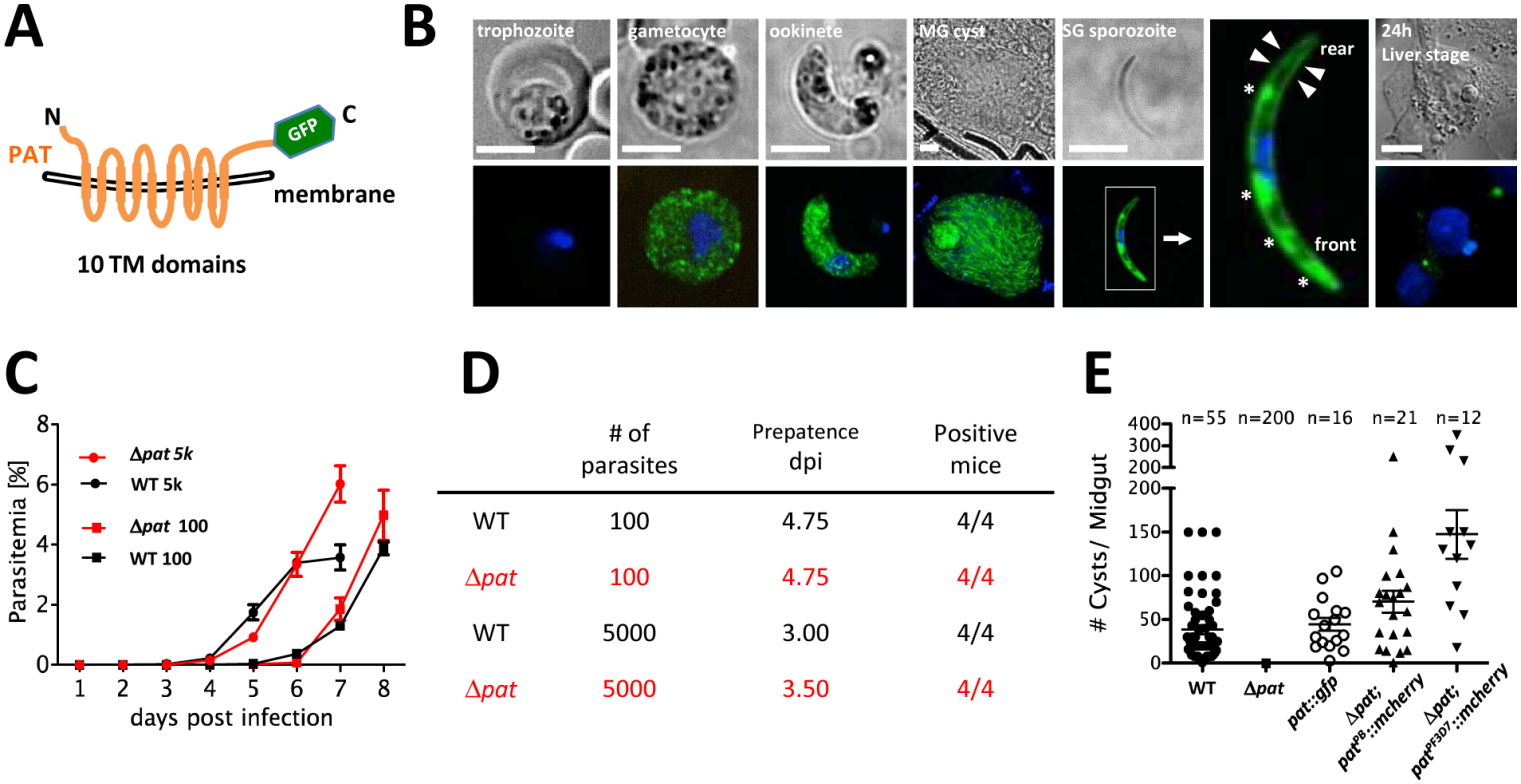
PAT is expressed in transmission stage parasites and redundant for asexual development but essential for mosquito infection. (A) Predicted membrane topology of *P*. *berghei* PAT; the cartoon includes a C-terminal GFP tag. (B) Live cell imaging of *pat*::*gfp* parasites. In gametocytes and ookinetes PAT::GFP is distributed throughout the cell in a speckled, intracellular pattern, while it localizes to the plasma membrane (arrowheads) and micronemes (asterisks) in sporozoites. (C) Mice infected with either 5000 (5k) or 100 *pat* blood stage parasites develop parasitemia similar to wildtype. (D) Summary table of blood stage infection with prepatency period of parasites as determined in Giemsa-stained smears. (E) Oocyst numbers of wildtype and the following mutants: *pat; pat*::*gfp; pat;pat* ^*PB*^::*mcherry;pat; pat* ^*PF3D7*^::*mcherry;* parasites were quantified on days 12–14 after mosquito infection. Data mean ± SEM.

*P*. *berghei* PAT PBANKA_0303900 belongs to the major facilitator superfamily (MFS) of transporters and is a syntenic ortholog of the *P*. *falciparum* vitamin B_5_ transporter PAT PF3D7_0206200 [31]. The MFS protein family (Pfam:PF07690; Superfamily:SSF103473) is characterised by the presence of 10–12 transmembrane domains (Fig 1A) and is evolutionarily widely distributed from bacteria to plants and humans [30, 32]. Twelve such proteins are known in rodent and human malaria species (S1 Fig, www.plasmodb.org). The PBANKA_030390 protein in *P*. *berghei* is 541 amino acids long and highly conserved between the various malaria species with 97.2% identity (98.9% similarity) between proteins from the rodent species *P*. *berghei* and *P*. *yoelii*, and 75.9% identity (85.6% similarity) between proteins from *P*. *berghei* and *P*. *falciparum* despite a 30 amino acid asparagine-rich insertion (S2 Fig). Outside the genus *Plasmodium* the nearest neighbours are found in related apicomplexans including *Toxoplasma gondii*, *Babesia* and *Eimeria* (S3 Fig). In global proteomic screens PAT has been identified in *P*. *berghei* sporozoites [33], and the *P*. *falciparum* ortholog PF3D7_0206200 was detected in stage V gametocytes [34] as well as salivary gland sporozoites [35] but not asexual stage parasites.

We had identified *pat* in a transcriptome analyses of sporozoites prematurely differentiating into exoerythrocytic (EEF)-like forms [36]. These mutants experience a slow loss of transcripts encoding gliding motility and invasion factors. The progressive downregulation of *pat* during transformation together with a cohort of 25 genes, which included *gap45*, *celtos*, *spect2*, *spect1*, *psop9*, *trep*, *siap1*, *mtrap* and *gest* (S4 Fig) suggested a role for PAT in motility or host cell invasion.

In order to assess the expression profile of *pat* we performed Reverse Transcriptase-PCR using life cycle stage-specific cDNAs. We found clear transcriptional evidence in sporozoite but not liver stage parasites. Transcription was also evident in mixed blood stage parasites containing asexuals and gametocytes as well as oocysts (S5 Fig) consistent with RNAseq data [37]. Next we tagged the endogenous *P*. *berghei* protein *in situ* with a C-terminal GFP-tag leaving the gene under the control of its own promoter in this haploid protozoan (S6 Fig). In the *pat*::*gfp* clonal line we found no expression in asexual blood stage parasites, although in *P*. *falciparum* the tagged protein was identified to be expressed in schizonts in a recent study [31]. *P*. *berghei* sexual precursor cells (gametocytes) on the other hand displayed strong fluorescence with a speckled appearance distributed throughout the cytoplasm akin to osmiophilic body staining [10–12, 14] (Fig 1B). A similar speckled expression pattern emerged in ookinetes, the motile parasites penetrating the mosquito midgut. In salivary gland sporozoites PAT::GFP localised to the apical and posterior ends of the parasite reminiscent of micronemes but was also clearly visible at the periphery, possibly the plasma membrane, where it was identified in a proteomic study of surface-exposed proteins of *P*. *falciparum* sporozoites [38]. Late liver stage forms did not produce any fluorescent cells consistent with microarray [36] and our RT-PCR data here.

### Δ*pat* parasites fail to establish mosquito infections

In order to address the role of PAT during life cycle progression of *P*. *berghei* in the mosquito we generated a knockout mutant named Δ*pat* (S7 Fig). Although generation of a gene deletion mutant had failed in a recent *P*. *falciparum* study [31] we readily established clonal knockout lines showing that in *P*. *berghei* PAT is redundant for blood stage development. The asexual developmental cycle proceeded with growth rates (Fig 1B) and prepatencies (Fig 1C) as wildtype parasites. Δ*pat p*arasites however failed to establish mosquito infections with no evidence for oocyst formation (Fig 1D) consistent with a *P*. *yoelii* [39] and a recent *P*. *berghei* study [40].

As expected, the Δ*pat* phenotype of *P*. *berghei* was rescued by mCherry-tagged *P*. *berghei* PAT in the line Δ*pat; pat* ^*PB*^::*mcherry* (S8 Fig) corroborating that the null mutant phenotype was due to the deletion of the *pat* gene. Although the *P*. *falciparum* protein was shown to transport vitamin B_5_ when expressed in yeast, *P*. *falciparum* PAT (S9 Fig), when introduced and expressed in the *P*. *berghei* Δ*pat* mutant, rescued the null mutant phenotype in the parasite Δ*pat; pat* ^*PF3D7*^::*mcherry*. Complementation with *P*. *berghei* and *P*. *falciparum* PAT restored mosquito infection (Fig 1D) suggesting a conserved role of PAT from human and rodent malaria species during mosquito infection.

### Δ*pat* male gametocytes present a defect in the formation of individual, free gametes

**Fig 2:**
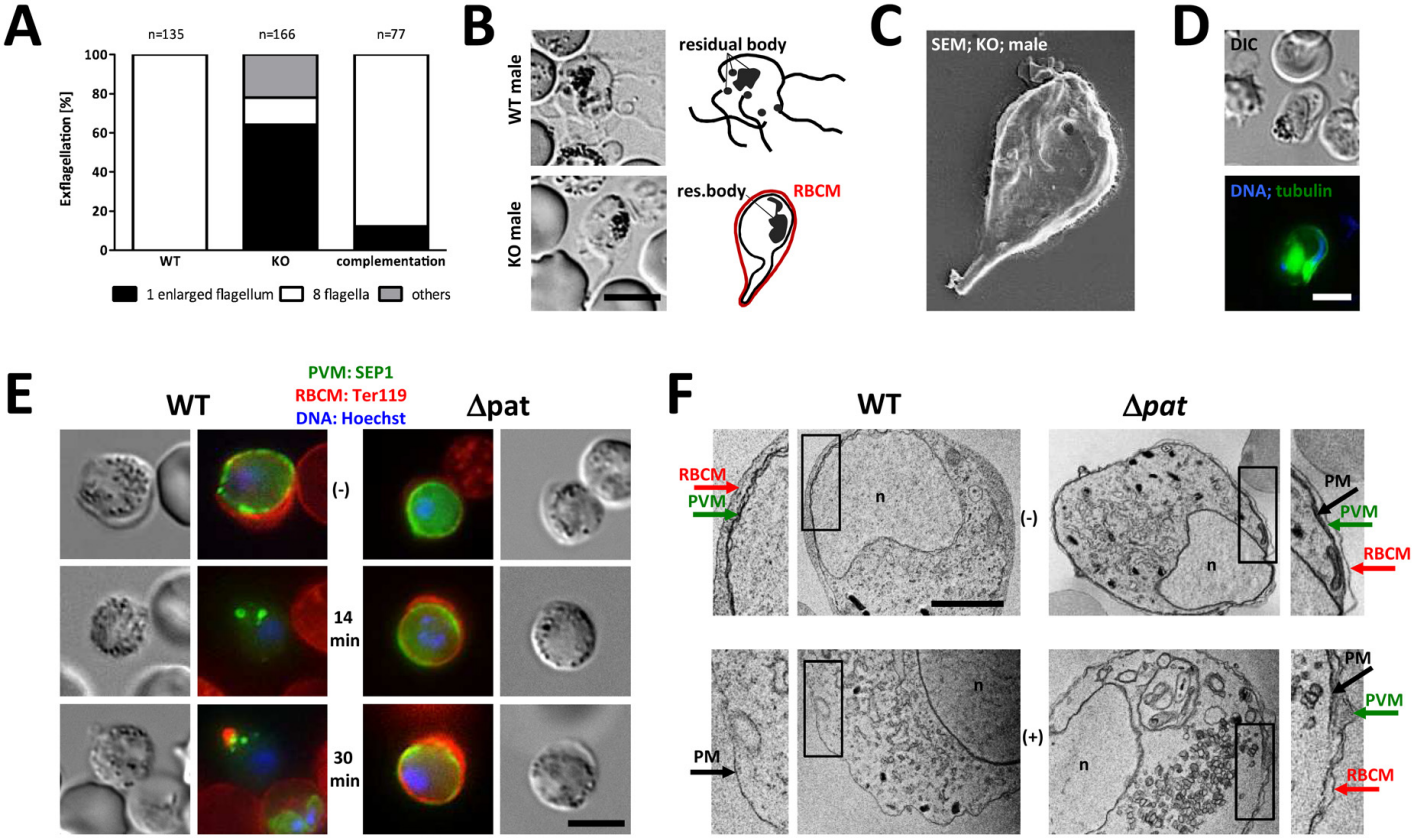
Male and female *pat* null mutant gametes suffer egress defects. (A) Quantification of exflagellation phenotypes in male gametes of wild type, Δ*pat* and *P*. *berghei* complementation parasites. Wild type microgametes form 8 individual flagella, while *pat* null mutants remain intraerythrocytic. Scale bar: 5 μm. (B) DIC images of wild type and Δ*pat* microgametes. Scale bar: 5 μm. (C) SEM image of Δ*pat* intraerythrocytic microgamete. (D) Trapped microgametes have undergone nuclear division and formed flagella as evidenced by tubulin staining. Scale bar: 5 μm. (E) IFA time-course of female wild type and Δ*pat* gametocytes before (-) and 14 to 30 minutes post activation. PVM (green) is stained with anti-SEP1; the RBCM (red) with anti-Ter119. Wildtype cells egress 14 minutes after activation was induced while Δ*pat* parasites remain still trapped 30 minutes post activation. Scale bar: 5 μm. (F) TEM images of female wild type and Δ*pat* gametocytes before (top row) and after activation (bottom row). Scale bar: 1 μm. Insets 200% enlarged. n, nucleus.

The non-infectious nature of Δ*pat* mutants suggested a critical role for PAT in gametogenesis, fertilization or ookinete formation. We first evaluated the exflagellation behavior of mutant male gametes. Wildtype male gametogenesis entails the formation of eight motile microgametes, which are easily recognised as vigorously beating flagella when observed under the microscope (S1 Movie). More than 60% of Δ*pat* parasites failed to egress from the RBC following activation, while wildtype cells never showed this defect (Fig 2A). The entrapment of elongated, flagellated male gametes within the RBC produced characteristic, tadpole-like RBC deformations that are evident in light (Fig 2B) and scanning electron microscopy (Fig 2C). Despite failing to emerge from the RBC, Δ*pat* males had undergone DNA replication as evidenced by the presence of individual nuclei, and assembled flagella demonstrated by tubulin-staining of fixed cells (Fig 2D). Although enclosed within the RBC, these mutants were motile (S2 Movie). This male exflagellation defect was rescued by *P*. *berghei* PAT::mCherry in a complementation experiment, where less than 10% showed the trapped males we had observed in Δ*pat* parasites (Fig 2A). Kooij *et al*. identified a 75% reduction in *P*. *berghei* exflagellation events [40], while in *P*. *yoelii* the defect was 40% [39].

### Δ*pat* female gametes fail to egress from PVM and RBC

We next investigated female gamete egress and found that they too did not emerge from the RBC. While wildtype cells rapidly dissolved the PVM (SEP1-labelled) [41] as well as the RBCM (TER119-labelled), Δ*pat* mutants dissolved neither membrane as late as 30 minutes after activation (Fig 2E). The failure to lyse these membranes could also be observed in TEM and revealed a tight, triple membrane structure in non-activated but also activated mutant gametocytes (Fig 2F). The failure of male and female mutant gametes to emerge from the RBC explained the lack of oocysts found in transmission experiments. While we failed to identify ookinetes in three independent *in vitro* experiments, the reduction in a recent study in *P*. *berghei* was 90% and 30% in *P*. *yoelii*; neither study reported the formation of oocysts [39, 40].

### PAT is a component of egress vesicles inhabited by G377 and PPLP2

**Fig 3:**
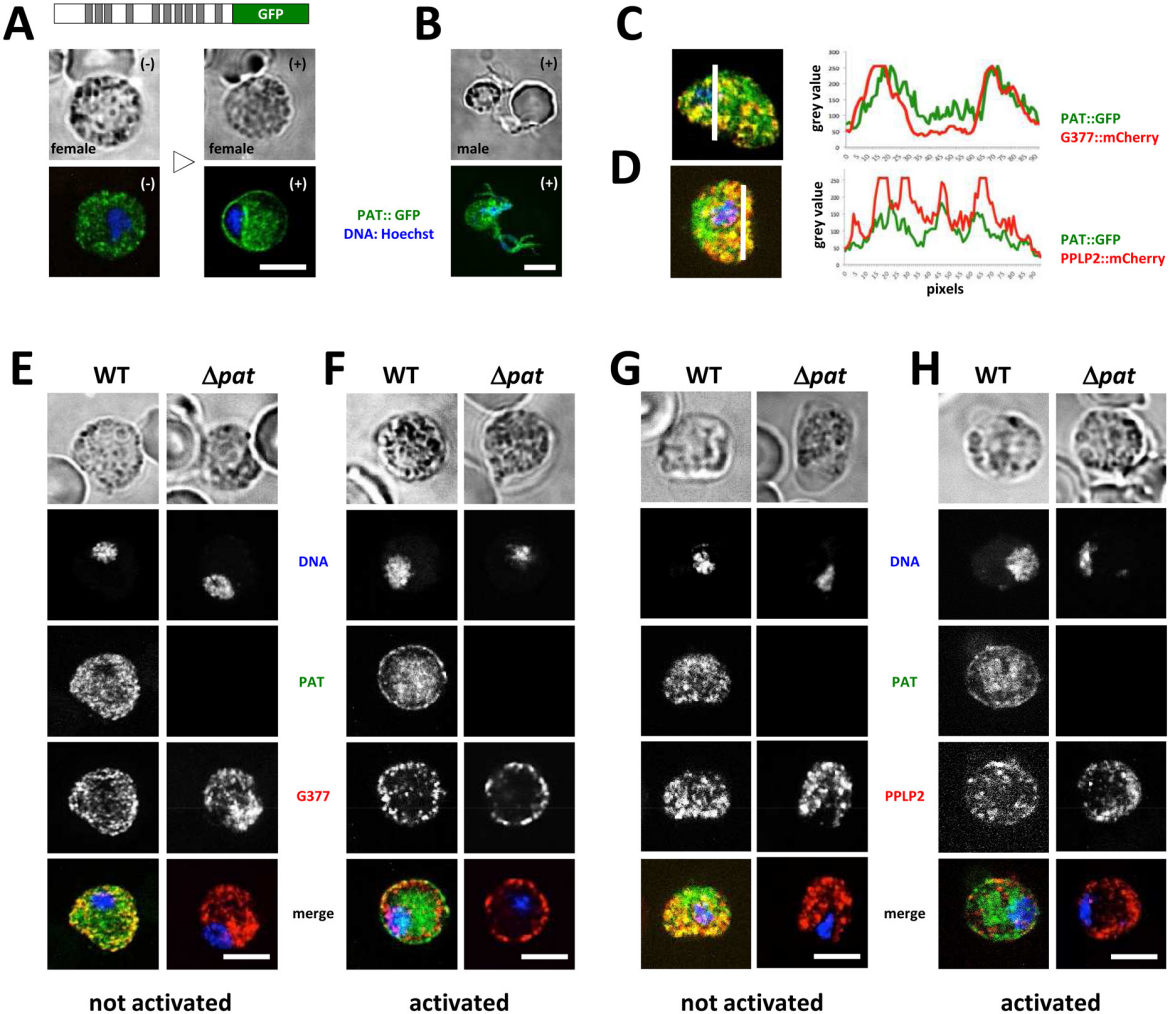
*P*. *berghei* PAT localizes to osmiophilic bodies (OB) but is not required for OB formation or trafficking. (A) PAT::GFP (model drawn to scale and indicating transmembrane domains in grey) traffics to the parasite plasma membrane upon activation in females. Scale bar: 5 μm. (B) PAT::GFP expression in exflagellating microgametes. Scale bar: 5 μm. (C) Female gametocyte expressing PAT::GFP and G377::mCherry. Co-localization was analyzed on white line using ImageJ. Scale bar: 5 μm. (D) Female gametocyte expressing PAT::GFP and PPLP2::mCherry. Co-localization was analyzed on white line using ImageJ. Scale bar: 5 μm. (E) G377::mCherry expression is unaltered in non-activated wildtype (WT) and Δ*pat* parasites. Scale bar: 5 μm. (F) G377::mCherry expression levels and trafficking of G377+ vesicles to the plasma membrane proceeds normal in activated wildtype (WT) and Δ*pat* parasites. Scale bar: 5 μm. (G) PPLP2::mCherry expression is unaltered in non-activated wildtype (WT) and Δ*pat* parasites. Scale bar: 5 μm. (H) PPLP2::mCherry expression levels and trafficking of G377+ vesicles to the plasma membrane proceeds normal in activated wildtype (WT) and Δ*pat* parasites. Scale bar: 5 μm.

Egress of males and females requires the concerted release of factors that together facilitate rupture of the PVM and the RBCM. Our data suggested a failure to produce or release egress molecules. In order to investigate the potential relationship between egress vesicles and PAT we first characterised the *pat*::*gfp* mutant and determined the GFP fusion protein’s localization in gametocytes and gametes in greater detail. We had revealed cytoplasmic speckles in male and female blood stage gametocytes indicative of a vesicular localisation and reminiscent of OBs (Fig 3A and 3B). Following activation of female gametocytes, we found PAT::GFP to distribute towards the plasma membrane (Fig 3A), suggesting a trafficking event of PAT-defined vesicles towards the cell surface similar to that observed in *P*. *falciparum*, where G377-positive (G377+) OBs are trafficked to the plasma membrane and secreted following gametocyte activation and gamete formation [9, 14].

To appreciate whether PAT labels G377+ OBs we generated the parasite line *pat*::*gfp;g377*:*mcherry* (S10 Fig) expressing G377::mCherry in the *pat*::*gfp* genetic background. This parasite line revealed widespread co-localization between PAT::GFP and G377::mCherry (Fig 3C): the majority of G377+ vesicles are PAT::GFP+ thus identifying this transporter as a component of OBs, where the protein most likely resides within the membrane virtue of 10 transmembrane domains. The presence of additional PAT+ but G377-negative (G377-) vesicles suggests that additional vesicles defined by PAT exist that are distinct from OBs containing G377. While G377, GEST and MDV1/PEG3 have previously been shown to reside in OBs [10, 12, 14], PPLP2 was suggested to inhabit a separate compartment [16]. A co-transfection resulting in the clonal line *pplp2*::*mCherry;pat*::*gfp* (S11 Fig) suggested that PPLP2 as well resides in vesicles defined by the membrane protein PAT (Fig 3D).

### Formation of secretory egress vesicles is not affected in *pat* null mutants

In order to understand whether PAT plays a role in the formation of vesicles or packaging of assigned proteins such as G377 or PPLP2 we generated parasite lines expressing G377::mCherry (S12 Fig) or PPLP2::mCherry (S13 Fig) in the Δ*pat* genetic background resulting in lines Δ*pat;g377*::*mcherry* and Δ*pat;pplp2*::*mcherry*. In both double mutants G377+ as well as PPLP2+ vesicles were abundant (Fig 3E and 3G) indicating that neither biosynthesis nor incorporation of the two protein factors into vesicles relied on the presence of the PAT transporter. During female gametogenesis G377+ (Fig 3F, S14 and S15 Figs) as well as PPLP2+ vesicles (Fig 3H) were trafficked normally to the plasma membrane in the null mutant background.

### PAT is essential for the formation of fusion-competent egress vesicles

**Fig 4:**
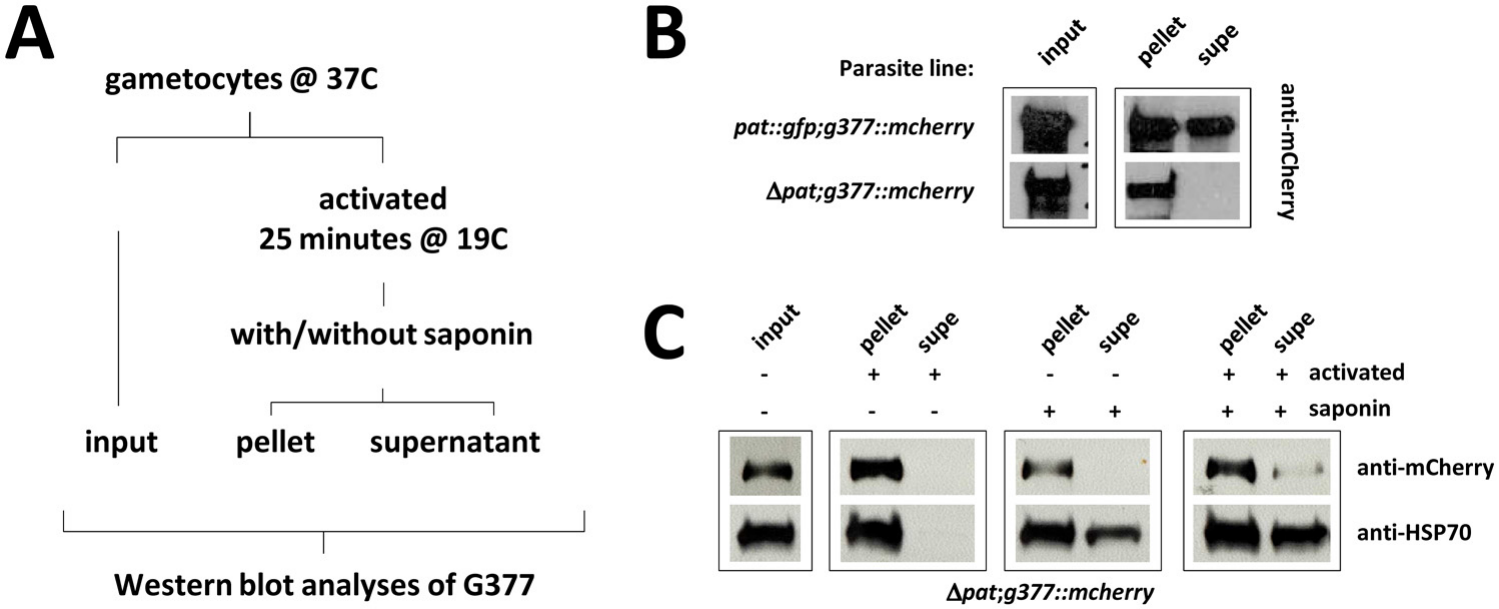
PAT is required for G377 osmiophilic body secretion. (A) G377 secretion assay approach. (B) G377::mCherry cannot be detected in egress supernatants of *pat* null mutants. (C) In *pat* null mutants G377::mCherry is not secreted into the PV space. HSP70 was used as a saponin lysis control.

We next developed an assay where G377 was used as a marker for egress vesicle secretion in female gametocytes (Fig 4A). Following gametocyte activation, we compared G377 secretion in the extracellular medium of the two parasite lines *pat*::*gfp;g377*::*mcherry* and Δ*pat;g377*::*mcherry* (Fig 4B). As shown before, neither PVM nor RBCM were lysed in the Δ*pat* null mutant background. As expected G377::mCherry could not be detected in the supernatant of activated Δ*pat;g377*::*mcherry* while the protein was abundant in the supernatant of the *pat*::*gfp;g377*::*mcherry* ‘wildtype’ background.

To exclude the possibility that G377 secretion had occurred in the Δ*pat* line but could not be detected as neither PVM nor RBCM were lysed—and thus remained within the PV space—we performed the following control experiment: after activation, both PVM and RBCM were artificially permeabilized with the detergent saponin without harming the plasma membrane of the parasite [42, 43]. HSP70, which is exported into the RBC cytoplasm [44], was used as control for efficient lysis and was indeed released and detected upon saponin treatment, but not before. G377 on the other hand was barely detectable in the supernatant (Fig 4C). This result shows that G377-containing vesicles are not secreted in the Δ*pat* null mutant. With the failure of null mutants to egress perhaps other egress vesicles defined by PAT are likely affected in the same manner precluding PVM and RBCM lysis and therefore gamete egress resulting in a total block of parasite transmission to the mosquito vector.

### In *P*. *berghei* sporozoites PAT localises to TRAP-positive micronemes

**Fig 5:**
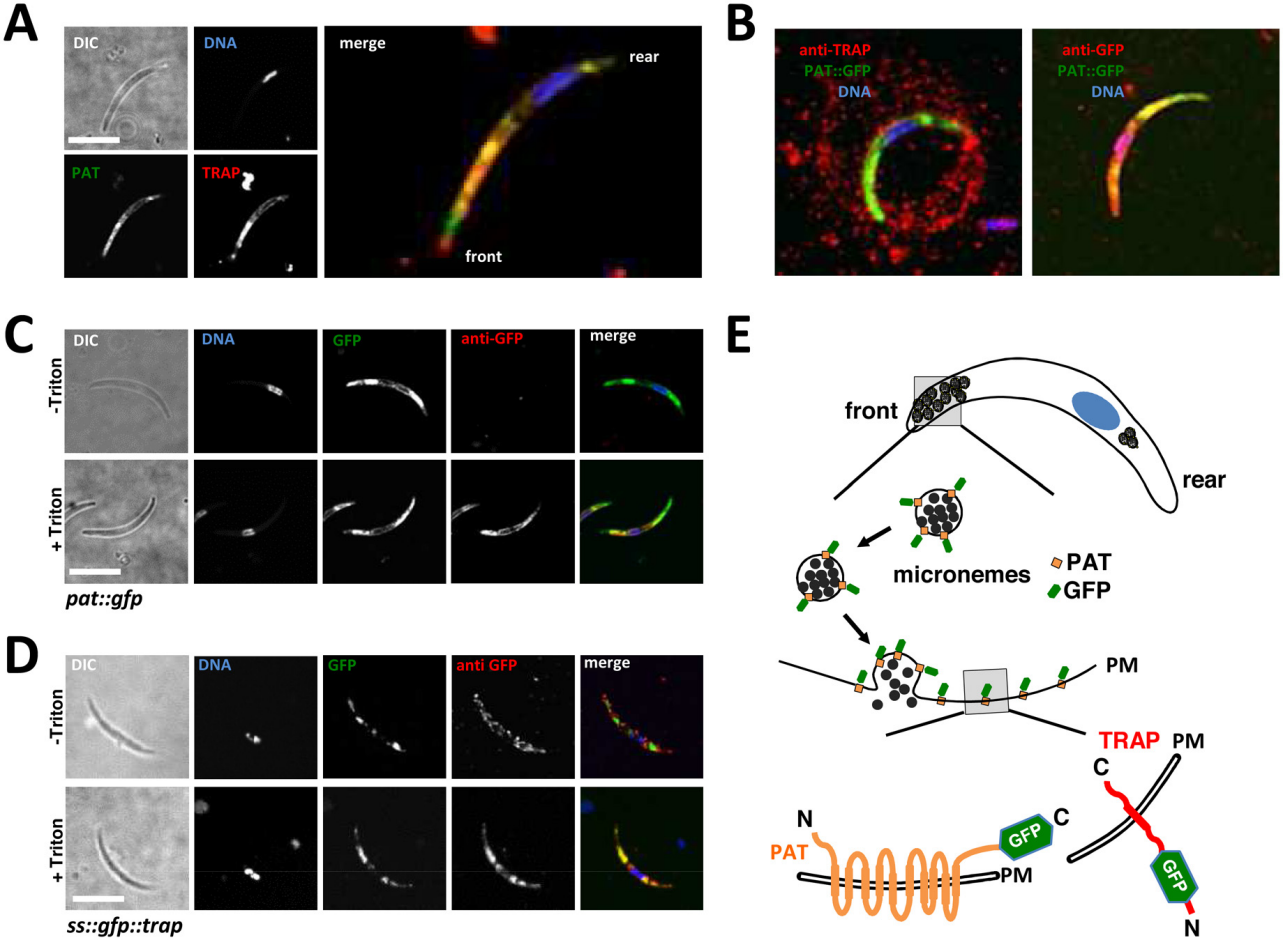
PAT::GFP is expressed in transmission stage parasites and co-localizes with TRAP. (A) Immunofluorescence staining of *pat*::*gfp* sporozoites with a TRAP-specific antibody reveals co-localization of TRAP with PAT::GFP. (B) While TRAP is secreted during gliding motility, PAT::GFP was not detected in trails. (C) IFA of PAT::GFP on permeabilized (+ Triton) or non-permeabilized (- Triton) sporozoites upon activation. (D) IFA of SS::GFP::TRAP on permeabilized (+ Triton) or non permeabilized (- Triton) sporozoites upon activation. (E) Schematic model of microneme secretion in sporozoites and proposed membrane topology of PAT::GFP compared to SS::GFP::TRAP. All scale bars: 5 μm.

Having established a clear role for PAT in egress vesicle secretion, we next wanted to detail the protein’s role in the sporozoite; there it is abundantly expressed in vesicular structures and present on the surface (Fig 1A) consistent with the recent identification of PAT by surface biotinylation of sporozoites [38]. In order to define PAT localisation in greater detail we performed co-immunostaining with the micronemal protein TRAP. This assay produced widespread intracellular co-localisation of TRAP with PAT::GFP (Fig 5A) supporting the suspected micronemal localisation. With 10 predicted transmembrane domains the protein is probably embedded into the membrane of these secretory vesicles and remains associated with the plasma membrane following secretion of micronemes. During gliding motility the type I transmembrane domain protein TRAP is discharged from micronemes and deposited in the plasma membrane; the C-terminal tail contacts actin filaments while the extracellular N-terminus provides attachment to the substrate; TRAP is finally translocated towards the back and cleaved by a rhomboid protease [26, 27]. Cleaved TRAP can therefore be visualised on microscope slides as characteristic circular trails when stained with antibodies (Fig 5B). PAT::GFP on the other hand did not localize in trails.

**Fig 6:**
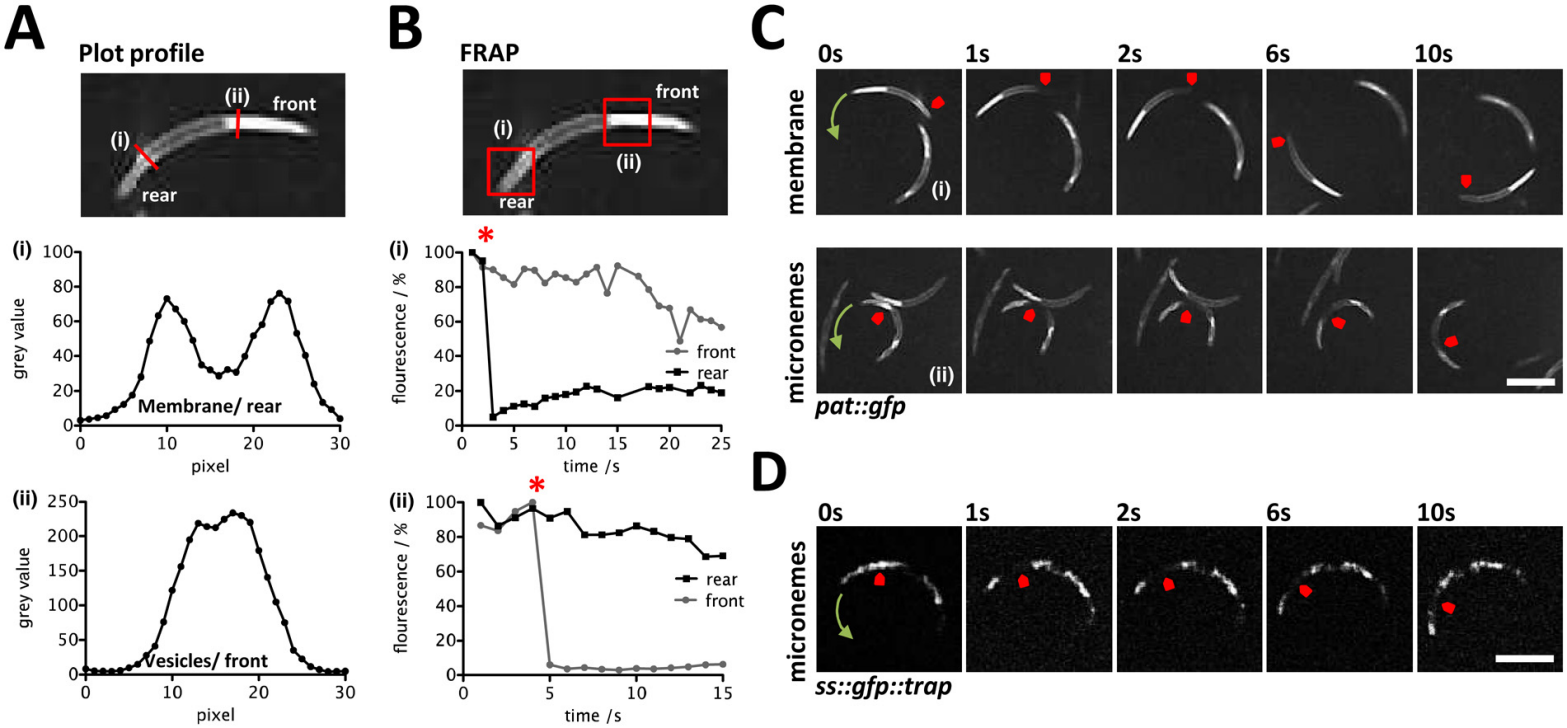
FRAP reveals dynamic mobility of membrane-bound PAT::GFP. (A) Plot profile along the red lines confirms membrane localization of PAT::GFP at the rear of the sporozoite (i) and the dominant microneme localization at the front of the sporozoite (ii) (B) Quantification of GFP signal recovery after FRAP at the membrane (i) and micronemes (ii), indicated in the red squares. (C) Representative images of sporozoites expressing PAT::GFP during FRAP at the membrane (i) and micronemes (ii). (D) Micronemal SS::GFP::TRAP does not recover after photobleaching. Arrows indicate direction of movement. All scale bars: 5μm.

In order to detail the membrane topology of PAT::GFP we performed immunofluorescence assays (IFA) in the presence and absence of a membrane-permeabilizing agent Triton X-100. In the absence of Triton X-100 anti-GFP antibodies failed to detect the protein in the *pat*:*gfp* line while cells treated with Triton X-100 produced a clear staining (Fig 5C). This would mean that the C-terminus of plasma membrane and microneme-embedded PAT is facing the cytoplasm (Fig 5D). A control experiment using a parasite line expressing N-terminally GFP-tagged TRAP (**SS::GFP::TRAP**; S16 Fig) showed a faint, speckled surface signal in non-permeabilized sporozoites (Fig 5E) consistent with earlier observations [26, 45] and which was noticeably different from the uniform membrane decoration by PAT::GFP. Plot profiles confirmed the peripheral localisation of PAT::GFP which was best seen in posterior regions of the parasite devoid of micronemes (Fig 6A). After photobleaching 25% of membrane-localized PAT::GFP recovered within ten seconds (Fig 6B(i) and 6C(i)); when considering the steady bleaching at the front of the parasite without FRAP, this figure rises to more than 40%. This dynamic mobility of membrane-bound PAT::GFP suggested translocation during gliding motility and a capacity to diffuse in the plasma membrane. The PAT::GFP signal of micronemes on the other hand did not recover at all, indicating that vesicles once generated are not exchanging proteins with other vesicles (Fig 6B(ii) and 6C(ii)). This was also found with the microneme signal of SS::GFP::TRAP (Fig 6D).

### Promoter swap supports mosquito infection but generates a defect in salivary gland invasion

The failure of the Δ*pat* mutant to establish oocysts and produce infectious sporozoites precluded the analysis of null mutant parasites during the sporozoite life cycle stage. In order to circumvent this transmission block we generated a mutant expressing PAT under the control of a *P*. *berghei* promoter that would support mosquito infection and the development of sporozoites, but which would ultimately be silent in mature sporozoites thus producing functional sporozoite gene knockouts. We chose the PBANKA_1300700 *ccp* promoter that has experimentally been shown by promoter GFP constructs and *in situ* tagging to be active in gametocytes and ookinetes but silent in late oocysts and sporozoites [46]. Using double-crossover homologous recombination, we introduced a plasmid into the *pat* locus resulting in the complete substitution of the endogenous *pat* promoter with the *ccp* promoter resulting in the parasite line *pat* ^*ccp*.*PP*^ (S17 Fig).

**Fig 7:**
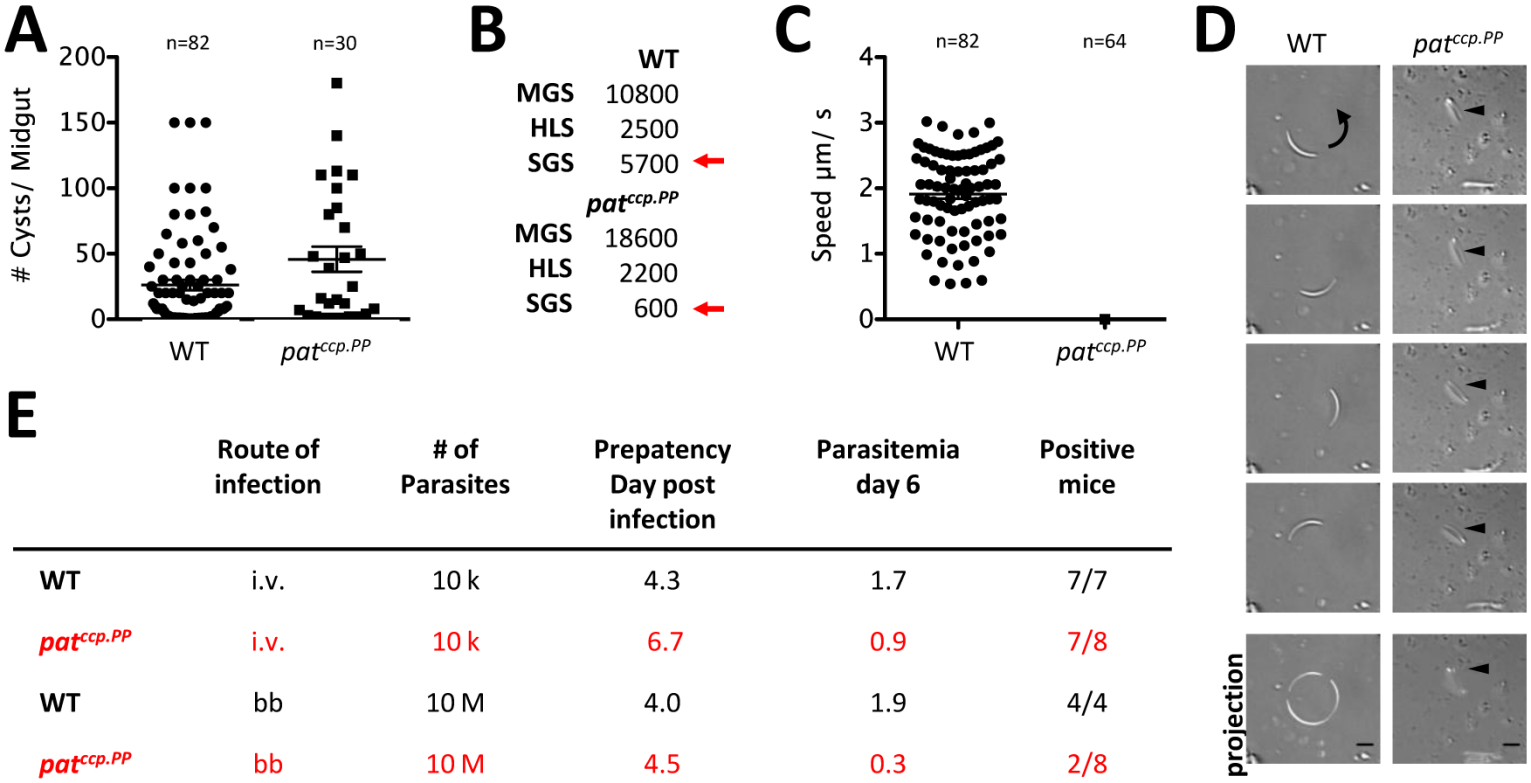
Expression of *pat* under the *ccp* promoter leads to the development of immotile and infection-deficient sporozoites. (A) Oocyst numbers of wildtype and *pat* ^*ccp*::*PP*^ collected on days 12–14 after mosquito infection. (B) Infection rates of sporozoites from midgut (MGS), hemocoel (HLS) and salivary glands (SGS) infected with either wildtype (WT) or *pat* ^*ccp*::*PP*^ show reduced salivary gland infections in the mutant. (C) Quantification of speed of sporozoites. (D) DIC live cell imaging of sporozoites. Arrow indicates direction of movement. Scale bar: 5μm. (E) Table summarising number of infected mice and respective prepatency periods after i.v. and bite-back (bb) infections. Data mean ± SEM.

Following the failure of Δ*pat* to produce oocysts in *P*. *berghei* here and elsewhere [40] but also in *P*. *yoelii* [39] we first compared the infection behaviour of the promoter swap mutant. Parasite transmission into mosquitoes in a standard feeding assay resulted in the establishment of *pat* ^*ccp*.*PP*^ oocysts in the range of wildtype infections (Fig 7A). The large number of midgut sporozoites in the *pat* ^*ccp*.*PP*^ mutant however did not translate into highly infected salivary glands, which was not due to a failure of the mutant to exit the oocysts and enter the hemolymph (Fig 7B).

### *pat* ^*ccp*.*PP*^ mutants are defective in adhesion, gliding motility and transmission

To determine whether PAT is required for sporozoite motility we isolated salivary glands from infected mosquitoes and performed gliding motility assays on microscope slides [27]. Under these conditions wildtype sporozoites glide in a circular fashion with a speed of 1–3 μm.s^-1^ in the presence of bovine serum albumin. *pat* ^*ccp*.*PP*^ mutant sporozoites on the other hand were immotile (Fig 7C and 7D).

Upon intra-venous (i.v.) injection of 10,000 salivary gland sporozoites, the parasitemia of mice injected with *pat* ^*ccp*.*PP*^ parasites reached on average 0.9% at day 6 (n = 8) in contrast to mice infected with wildtype sporozoites, which on average had about 1.7% (n = 7). Similarly, in mice infected by mosquito bite (ten infected mosquitoes were allowed to blood feed for 15 minutes on a naïve mouse), *pat* ^*ccp*.*PP*^ reached on average 0.3% parasitemia over the same period (n = 2; with 6 out of 8 mice remaining entirely parasite-free), while wildtype parasites reached 1.9% (n = 4). These data showed that Δ*pat*-infected mice had consistently lower parasite burdens during infection (Fig 7E). When sporozoites were i.v. injected into mice there was no difference in the number of infected mice yet in both experiments we observed a significant delay of Δ*pat* in prepatency (the first day at which parasites can be detected on Giemsa stained blood smears). The failure to efficiently establish blood stage infections and the delay in prepatency after mosquito bite and i.v. infections of mice identifies a role for PAT in motility, which is required during salivary gland entry, skin passage, cell traversal and invasion of host hepatocytes. We excluded a role of the protein during liver stage development since according to our RT-PCR data it was not expressed. Due to the reduced amount of mature sporozoites in salivary glands of the mosquito we never had enough parasites to reliably address this question.

### Conserved function of *P*. *falciparum* PAT in the mosquito vector independent of pantothenic acid transport

**Fig 8:**
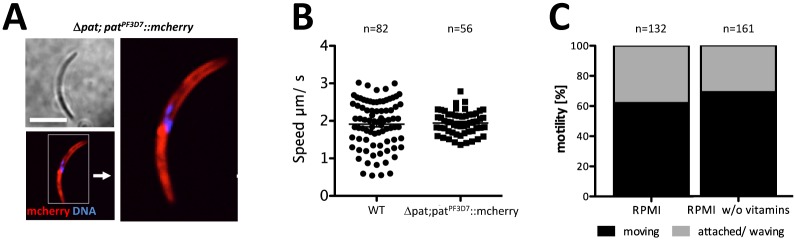
Conserved function of *P*. *falciparum* PAT—gliding motility is not influenced by pantothenic acid (vitamin B_5_). (A) Localization and expression of *P*. *falciparum* PAT::mCherry in the *pat* background. Scale bar: 5μm. (B) Quantification of speed of wildtype (WT) and PF3D7 PAT-expressing sporozoites. (C) Quantification of gliding motility of wildtype sporozoites in the presence or absence of pantothenic acid. Data mean ± SEM

In the human malaria parasite *P*. *falciparum* the PAT ortholog has been described as a vitamin B_5_ transporter essential for blood stage development [31]. This essential blood stage function is not conserved in *P*. *yoelii* [39] nor *P*. *berghei* (our data and [40]) and complementing our Δ*pat* line with PF3D7 PAT::mCherry resulted in the normal formation of oocysts while Δ*pat* failed to do so entirely. Δ*pat* sporozoites stably expressing PF3D7 PAT::mCherry showed a localization pattern identical to PAT::GFP (Fig 8A). When we tested gliding motility behaviour of *pat; pat* ^*PF3D7*^::*mcherry* parasites we found a gliding motility speed comparable to wildtype (Fig 8B) while promoter swap mutants *pat* ^*ccp*.*PP*^ were immotile (Fig 7C).

In order to test whether pantothenate (vitamin B_5_) is required for motility of wildtype *P*. *berghei* we assayed gliding in the absence and presence of vitamin B_5_ (0.25 mg/ml). Both standard medium as well as the one lacking vitamin B_5_ supported normal parasite motility; an immediate exogenous source for pantothenate is thus not required for the duration of the sporozoite gliding motility assay (Fig 8C).

### SS::TRAP::GFP expressed in Δ*pat* sporozoites reveals a microneme secretion defect

**Fig 9:**
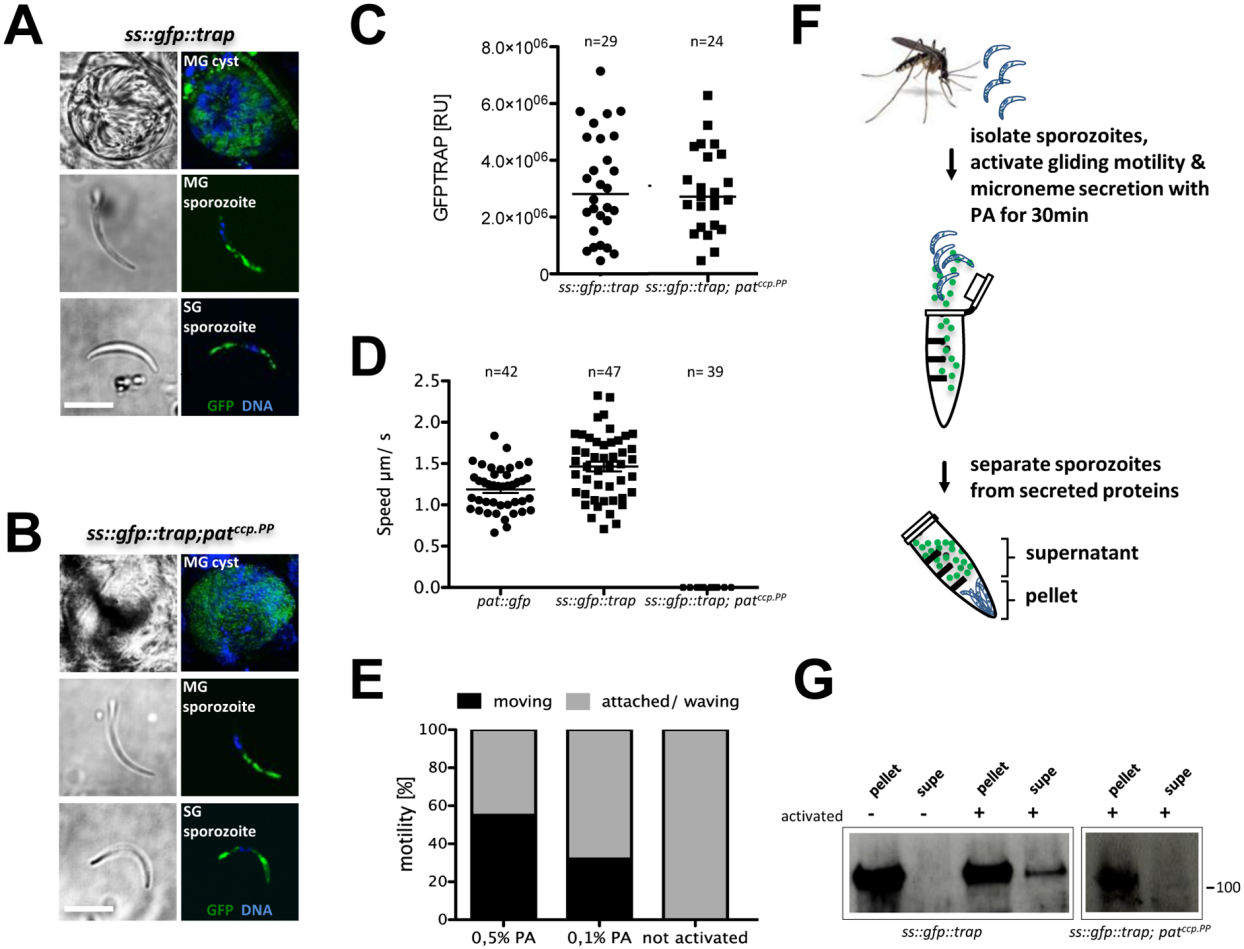
PAT is required for microneme secretion. (A) Localization of SS::GFP::TRAP in wildtype background sporozoites. (B) Localization of SS::GFP::TRAP in *pat* ^*ccp*.*PP*^ background sporozoites. (C) Quantification of SS::GFP::TRAP fluorescence intensity in wildtype and mutant. (D) Quantification of speed of sporozoites. (E) Quantification of gliding motility of wildtype sporozoites activated with pluronic acid (F) Model of microneme secretion assay developed for TRAP detection by Western blot. (G) Western blot of 25k sporozoites of SS::GFP::TRAP in wildtype and promoter swap mutant using anti-GFP antibodies. Scale bars: 5μm. Data mean ± SEM.

To investigate whether microneme formation or its discharge in the immotile *pat* ^*ccp*.*PP*^ parasites was affected, we first developed a parasite line expressing an additional copy of TRAP tagged with GFP sandwiched between the signal sequence and the A-domain in the *pat* ^*ccp*.*PP*^ background (S18 Fig). The mutant line *ss*::*gfp*::*trap;pat* ^*ccp*.*PP*^ allowed us to determine whether TRAP expression, trafficking into micronemes or secretion was affected in the absence of PAT. In both the wildtype (Fig 9A) and mutant background (Fig 9B) SS::GFP::TRAP is expressed in midgut and salivary gland sporozoites with a localisation consistent with wildtype TRAP.

Comparing expression levels by quantifying total fluorescence intensities of SS::GFP::TRAP in the wildtype background and the *pat* ^*ccp*.*PP*^ mutant showed no difference (Fig 9C). But while the *pat*::*gfp* and *ss*::*gfp*::*trap* mutants produced gliding sporozoites, the *ss*::*gfp*::*trap;pat* ^*ccp*.*P*^ mutant did not (Fig 9D).

The failure to glide was thus not a result of aberrant microneme formation, but could perhaps be found in a defect in microneme secretion. In order to address this possibility we developed a gliding motility assay based on the activation of sporozoites using pluronic acid (PA) instead of bovine serum albumin (BSA) to allow the detection of proteins by Western blot otherwise masked by excessive amounts of BSA. BSA activates a signaling cascade [47] leading to the mobilization of calcium and the secretion of micronemes [48, 49]. Pluronic acid (PA) also stimulates calcium influx into cells [50] and efficiently triggers activation of gliding motility in sporozoites (Fig 9E). A standard gliding assay with wildtype sporozoites using RPMI supplemented with 0.5% PA resulted in a percentage of moving cells similar to the wildtype.

As *ss*::*gfp*::*trap;pat* ^*ccp*.*PP*^ sporozoites do not adhere to the substrate, both wildtype and mutant sporozoites were activated—and stimulated to secrete adhesins—in suspension for 30 minutes. Parasites were then separated from the supernatant by centrifugation and the secreted proteins were analyzed by Western blot (Fig 9F). In the wildtype background SS::GFP::TRAP is secreted and cleaved in activated sporozoites. The GFP fusion protein produced a clear signal in the reference line *ss*::*gfp*::*trap*. However, this was absent in the *ss*::*gfp*::*trap;pat* ^*ccp*.*PP*^ mutant (Fig 9G) supporting our hypothesis of microneme secretion failure and thus absence of attachment and gliding motility in the promoter swap line, and thus explaining the similar phenotype found for the *trap* knockout.

## Discussion

Our results establish the transporter PAT from the rodent malaria parasite *P*. *berghei* as a central component of the secretory machinery of transmission stage parasites. The protein is part of the egress machinery of female and male gametes where it is required for the secretion of osmiophilic bodies and remarkably, PAT also localises to micronemes in the sporozoite and is required for TRAP secretion.

### PAT in gametocytes

Gene disruption of *pat* resulted in normal development of blood stage parasites but prohibited transmission to the mosquito vector (Fig 1) as has also been found in recent studies in *P*. *yoelii* and *P*. *berghei* [39, 40]. While these initial studies simply described the phenotype of a *pat* deletion, we went further to characterize in detail the functional involvement of PAT not only in gametocytes, but also in sporozoites using a promoter swap strategy.

This showed that Δ*pat* gametocytes, in particular females, generated normal numbers of G377+ OBs that trafficked to the plasma membrane after activation but that secretion of G377 into the PV space was entirely inhibited. This resulted in female gametocytes being trapped inside the RBC, unable to participate in fertilisation and normal developmental progression and ultimately a complete block in transmission to the mosquito vector (Fig 2). Although OBs are more abundant in females than in males [14] we have no reason to believe that secretion of egress vesicles is not affected in males. Like females they remain, albeit not entirely, trapped within the RBC. While the molecular mechanism is unknown, the failure of Δ*pat* parasites to egress from the RBC clearly lies in the inability of mutants to dissolve the PVM and RBCM (Fig 2). Likely depending on the secretion of multiple protein factors, we find that Δ*pat* mutants fail to secrete G377 during egress [11, 14], a protein found to co-localise with MDV1/PEG3 [14]. The MFS transporter does not affect egress vesicle formation, nor trafficking of G377+ OBs and PPLP2+ vesicles to the plasma membrane following gametocyte activation (Fig 3). However, PAT is clearly required for the fusion of these vesicles with the plasma membrane and the release of its contents into the PV space as shown in our saponin experiment (Fig 4). Whether additional egress factors suffer from the same defect is unknown, but can be expected. In *P*. *berghei* PAT thus acts as a defining membrane component of specialised secretory vesicles in this crucial life cycle stage of the parasite, the male and female gametocyte. PAT not only defines OB but also those containing the perforin PPLP2 (Fig 3). None of the known egress molecules (PPLP2, G377, GEST, MDV1/PEG3) by themselves are essential for egress [10–12, 14, 16, 51]. Only a combined failure to be secreted could result in the complete transmission block observed in Δ*pat* parasites, supporting PAT as an upstream, common factor with an essential function for OB biology rather than affecting individual protein content of such vesicles. Egress may even require additional, hitherto unknown factors. Our data support a clear molecular chain of events where OB factors like G377 are packaged into vesicles and trafficked to the plasma membrane of the parasite following a change in host environment; only then is PAT necessary for vesicle fusion allowing OB content to be released into the PV space promoting PVM and RBCM lysis. How PAT defines docking or fusion of egress vesicles at the parasite’s plasma membrane is unknown, and will rely foremost on a detailed biochemical comparison of egress vesicles isolated from wildtype and Δ*pat* mutants.

The *P*. *falciparum* PAT ortholog was reported to be an essential blood stage vitamin B_5_ transporter with different expression and localization profiles [31]. However, a *P*. *yoelii* and recent *P*. *berghei* knockout line of its *pat* ortholog showed no affect on asexual parasite development and instead failed to produce oocysts [39, 40]. These discrepancies are puzzling, but while not excluding the possibility that *P*. *berghei* PAT may transport vitamin B_5_ in a heterologous yeast system just like the *P*. *falciparum* protein, we find that the *P*. *falciparum* ortholog does complement the *P*. *berghei* Δ*pat* strain, rescuing the egress defects observed in males as well as females resulting in the successful formation of oocysts and mosquito infections. This complementation of the *P*. *berghei* knock out line with the *P*. *falciparum* gene strongly suggests that both proteins play similar functions during mosquito development of the parasite.

### PAT in sporozoites

In sporozoites microneme secretion is required for the positioning of adhesins to the parasite surface in order to provide a link between the acto-myosin motor with the extracellualr substrate [52]. Using a promoter-swap strategy to specifically deplete PAT from sporozoites we found that secretion of TRAP-containing vesicles requires PAT. *P*. *berghei* PAT constitutes the first membrane protein found with a function in microneme secretion. In the absence of PAT in sporozoites, TRAP secretion is defunct (Fig 9) leading to a parasite population with a 95% reduction in mosquito salivary gland invasion rates and a 75% decrease in re-infection of the rodent host (Fig 7). Similar to the Δ*trap* mutant [22], parasites lacking *pat* largely fail to adhere to the substrate and do not glide. Intriguingly, Δ*pat* sporozoites isolated from the hemolymph or salivary gland were not able to move in the typical back-and-forth manner as shown for Δ*trap* sporozoites [27], indicating that additional proteins are secreted in a PAT-dependent fashion that are important for this form of motility. However, when injected i.v. into the rodent host Δ*pat* sporozoites could establish an infection (Fig 7), unlike Δ*trap* sporozoites [22]. The observed 2-day delay in prepatency after i.v. injection suggests a key role for liver infection. Yet, the fact that some sporozoites could establish an infection also hints that Δ*pat* sporozoites might undergo some movement in 3D while they fail to attach to 2D substrates, as has been also seen in *P*. *berghei* sporozoites lacking the actin-binding protein coronin [53] or for *T*. *gondii* [54, 55]. Alternatively, small amounts of TRAP family proteins could also be secreted in a microneme-independent fashion.

In sporozoites PAT::GFP and TRAP co-label the same subcellular compartments and behave similarly in FRAP experiments (Fig 5). Mature vesicles localizing to the anterior of the parasite did not recover following photobleaching corroborating the notion that micronemes are formed directly from the Golgi [56] and accumulate unaltered at the apical part of the sporozoite until ready to be discharged. During microneme secretion both TRAP and PAT are deposited in the plasma membrane (Fig 5). However only TRAP displays localized, speckled protein foci while PAT presents a uniform membrane distribution and disperses freely in the plasma membrane. This difference might be due to the localization of TRAP to discrete adhesion sites [27, 57], while PAT might not have a localized function on the sporozoite surface. We found no difference in gliding whether vitamin B_5_ was present or not. Thus, PAT either has different functionalities at different life cycle stages of the *Plasmodium* parasite and does not operate as a pantothenate transporter in the sporozoite, or sporozoites had already stored enough pantothenate (or produced Coenzyme A) sufficient for gliding. In *P*. *falciparum pat* was presented as an essential blood stage gene capable of complementing—partially fused to yeast Fen2p—the yeast *fen2p* knockout line [31]. However, such an essential role could not be confirmed in *P*. *yoelii* or *P*. *berghei* where blood stage development occurred unabated in the absence of this protein [39, 40]. Using *in situ* GFP tagging of the endogenous gene expression in *P*. *berghei* was restricted to gametocytes and mosquito stage parasites (Fig 1). In accordance with the *P*. *yoelii* study our Δ*pat* mutant failed to transmit to the mosquito vector (Fig 1), which is consistent with the protein expression observed in this study and previous proteomic screens. Surprisingly however, this defect was rescued by the expression of the *P*. *falciparum* protein in the Δ*pat* line and also localized identically (Fig 1).

The detailed molecular mechanism by which PAT affects *P*. *berghei*—and most likely *P*. *yoelii*—transmission and motility and whether vitamin B_5_ is involved at this parasite stage remain to be elucidated. While the protein shows similarity with Fen2p (S19 Fig), there is also homology with the *Drosophila melanogaster* sphingosine 1-phosphate transporter Spinster (S20 Fig) [58], and several uncharacterized MFS transporters of higher plants (*Arabidopsis thaliana* for example) show even higher similarity (S21 Fig). Interestingly, in neurons the incorporation of sphingosine into synaptic vesicles promotes the interaction with plasma membrane localized SNARE proteins and exocytosis [59]. Our data support a clear function for the protein in defining the fusion competence of TRAP-containing micronemes. These vesicles are releasing their content after elevation of intracellular calcium [49]. Calcium signaling is activated by extracellular ligands such as serum albumin [47, 60] or small tripeptides [61]. Activated Δ*pat* sporozoites failed to secrete TRAP and hence we presume that PAT acts downstream of calcium signaling, possibly by playing a role in microneme docking to the plasma membrane.

Both osmiophilic body and microneme secretion are tightly regulated events, they require calcium-signalling under the right circumstances. It is intriguing to see that specialised secretory vesicles of fundamentally different life cycle stages require at least in part similar protein components for efficient secretion. We have shown here that *P*. *berghei* PAT a transporter belonging to the MFS superfamily defines fusion-competent egress vesicles, which in its absence are formed and packaged correctly, but not released, and show the involvement of PAT in microneme secretion. Through its essential role for mosquito infection, and key role in gliding motility and a mediator of microneme secretion we propose that this but also previously identified transporters like NPT1[62] could be used as a potential drug targets to inhibit parasite transmission of two different life cycle stages between mosquito and host.

## Materials and Methods

### Ethics statement

All animal work was performed according to European regulations in compliance with FELASA guidelines and regulations, and approved by the state authorities (Regierungspräsidium Karlsruhe) § 8 Abs. 1 Tierschutzgesetz (TierSchG) (Aktenzeichen 35–9185.81/G-3/11). 6–8 weeks old female NMRI mice (Janvier Labs) were used for rearing of the parasites and infection of mosquitoes.

### *pat*::*gfp* parasite line

A 2417 bp product encompassing the entire ORF of pat (1626 bp) as well as 771 bp of the 5’ UTR region was amplified from *P*. *berghei* genomic DNA with primers g1025 and g1027. The amplicon was cut with HincII and BamHI to release 1539 bp of the 3’ end of the ORF; this fragment was ligated into pLIS0010 (ΔNheI) containing the Toxoplasma dhfr/ts selection cassette to yield a C-terminal GFP fusion. The final plasmid pLIS0301 was digested with NheI, precipitated with 2.5 volumes ethanol and transfected into wildtype schizonts.

### Δ*pat* parasite line

A 744 bp fragment upstream of PBANKA_030390 (pat) (primers g0792/KpnI and g0793/HindIII), as well as a 836 bp fragment downstream of the stop codon (primers g0794/EcoRI and g0795/NotI) were PCR-amplified from *P*. *berghei* genomic DNA and cloned into b3D (mr4.org), either side of the *Toxoplasma gondii* dhfr/ts selection cassette. The final plasmid pLIS0076 was digested with KpnI and KspI, precipitated with 2.5 volumes ethanol and transfected into wt schizonts.

### Δ*pat;pat* ^*PB*^::*mcherry* parasite line

A 2313 bp fragment encompassing the entire ORF of *P*. *berghei pat* (PBANKA_030390) plus 672 bp of upstream region was PCR-amplified from wild-type genomic *P*. *berghei* DNA with primers g1220 and g1027 and cloned in frame with mCherry containing the human dhfr selection cassette. The final plasmid pLIS0201 was digested with EcoRV, precipitated with 2.5 volumes ethanol and transfected into Δ*pat* schizonts.

### Δ*pat;pat* ^*PF3D7*^::*mcherry* parasite line

A 1704 bp fragment encompassing the near-complete ORF *of P*. *falciparum* PAT PF3D7_0206200 (starting from amino acid 4) was PCR-amplified from *P*. *falciparum* PF3D7 genomic DNA with primers g3110 (BsmI) and g3111 (BamHI) and cloned into pLIS0201 resulting in an almost complete exchange of *P*. *berghei pat* for *P*. *falciparum* PF3D7_0206200; only the first three amino acids of the fusion gene remain from *P*. *berghei* (MNA), the remainder is *P*. *falciparum* sequence. The final plasmid pLIS0301 was digested with EcoRV, precipitated with 2.5 volumes ethanol and transfected into Δ*pat* schizonts.

### *pat*::*gfp;g377*::*mcherry* parasite line

A 1493 bp fragment encompassing the 3’ end of the ORF of *P*. *berghei* G377 (PBANKA_146300) ORF (starting from amino acid 4) was PCR-amplified from *P*. *berghei* genomic DNA with primers g3092 (PacI) and 3093 (BamHI) and cloned into pLIS0201 to create a C-terminal mCherry fusion. The final plasmid pLIS0244 was digested with BstZ17I, precipitated with 2.5 volumes ethanol and transfected into *pat*::*gfp* schizonts.

### Δ*pat*::*gfp;g377*::*mcherry* parasite line

pLIS0244 was digested with BstZ17I, precipitated with 2.5 volumes ethanol and transfected into Δ*pat* schizonts.

### *pat*::*gfp;pplp2*::*mcherry* parasite line

A 1441 bp fragment encompassing the 3’ end of the ORF of *P*. *berghei* PPLP2 (PBANKA_143240) ORF was PCR-amplified from *P*. *berghei* genomic DNA with primers g3103 (BstZ17I) and 3104 (BamHI) and cloned into pLIS0201 to create a C-terminal mCherry fusion. The final plasmid pLIS0281 was digested with PmeI, precipitated with 2.5 volumes ethanol and transfected into *pat*::*gfp* schizonts.

### Δ*pat*::*gfp;pplp2*::*mcherry* parasite line

pLIS0281 was digested with PmeI, precipitated with 2.5 volumes ethanol and transfected into Δ*pat* schizonts.

### *ss*::*gfp*::*trap* parasite line

To tag TRAP with GFP at the N-terminal after signal peptide cleavage, 1054 bp of the 5’UTR of *trap* together with the first 105 bp of the coding region were cloned in front of GFP linked by a 6-amino acid linker. This was followed by an 8-amino acid linker, the rest of the coding sequence of *trap*, followed by 514 bp of the 3’ UTR region of *dhfs*, followed by a *hdhfr* selection cassette. The pasmid was linearized with BstBI within the TRAP 5’UTR resulting in introduction of SS::GFP::TRAP by single crossover integration.

### *ccp* promoter swap parasite line

To change time and sex-specific MFS expression a generic plasmid was first generated that contains the following parts: 348 bp of 5’ UTR of PBANKA_030390 (PCR-amplified with primers 1144 and 1145), the *Toxoplasma dhfr/ts* selection cassette, followed by 543 bp of the 5’ end of the ORF of *pat* (PCR-amplified with primer 1148 and 1149). The promoter region from *ccp* (1273 bp) was then ligated immediately 5’ to the MFS ORF using the *Eco*RV and *Nde*I restriction sites from pL1260 [46] by *Ale*I and *Nde*I digestion. The plasmid was digested overnight with KpnI and KspI prior to transfection, precipitated and resuspended in AMAXA solution for nucleofector transfection of *P*. *berghei* schizonts; transfection, selection of mutant (drug-resistant) parasites and limiting dilution of clonal lines was as described [63].

### *Plasmodium berghei* parasite transfections

Transfection of respective schizonts was done by electroporation using the Amaxa nucleofector kit, followed by selection of mutant parasites with either pyrimethamine supplemented drinking water, or s.c. injection of WR99210 on four consecutive days and limiting dilution of clonal lines as described [19, 20]. Genotyping by PCR was performed as shown in Supplementary Figures.

### Live imaging of gametocytes

Imaging was performed with an Axiovert 200M microscope using either a 25x or 63x objective. One drop of infected tail blood was added to PBS containing Hoechst on a microscope slide and covered with a cover glass Exflagellation was imaged after induction of gamete formation by drop of temperature to 19°C for at least 10 minutes.

### Purification and activation of gametocytes

Blood of an infected mouse was harvested by cardiac puncture and gametocytes purified using a 49% Nycodenz cushion at 37°C (1500rpm; 20min). Cells were washed once with PBS and transferred to ookinete medium (250ml RPMI, HEPES, glutamine 12.5mg Hypoxanthine, 2.5ml Pen/Strep, 0.5g NaHCO_3_, 5.12mg Xanthurenic) at 19°C [64]. After indicated time points cells were either fixed with 4% PFA in PBS for immunostaining, or lysed in RIPA buffer for Western blot analyses.

### Immunofluorescence staining of gametocytes

After fixation, cells were permeabilized with 0.5% TritonX for 10 minutes. For visualization of the red blood cell membrane cells were incubated with anti-Ter119::Alexa488 (Biolegend 0.5mg/ml; ^1^/_1000_) antibody for 1h, washed twice with PBS, and resuspended in PBS containing Hoechst for observation under the microscope. For visualization of the parasitophorous vacuole (PV) membrane cells were incubated with anti-SEP1, followed by secondary anti-rabbit:: Alexa594 (Invitrogen 2mg/ml; ^1^/_1000_). Images were either taken on a Zeiss 200M Axiovert widefield (63x) or Nikon spinning disc (100x) microscope. Image processing was performed with ImageJ.

### Purification and activation of gametocytes

Blood of infected mice was harvested and gametocytes purified using a 49% Nycodenz cushion at 37°C (1500rpm; 20min). Cells were washed with PBS and transferred to ookinete medium (250ml RPMI, 12.5mg Hypoxanthine, 2.5ml Pen/Strep, 0.5g NaHCO3, 5.12mg xanthurenic) at 19°C [21]. After indicated time points cells were either fixed with 4% PFA in PBS for immunostaining, or lysed in RIPA buffer for Western blot analyses.

When RBC/PVM lysis was performed cells were put on ice for 2 min before adding 3μl of 10x Saponin (0.35%). After 30s on ice supernatant was collected, supplemented with protease inhibitors and used for analyses. Pellets were lysed in 60μl RIPA buffer. Detection of proteins was performed using the following antibodies: rabbit anti-mcherry antibody (abcam 1/5000), mouse anti-HSP70 obtained through the MR4 as part of the BEI Resources Repository, NIAID, NIH: *Mus musculus* b5, MRA-662, MF Wiser (1/20).

### Scanning electron microscopy

Cells were allowed to adhere to 0.1% poly-L-lysine-coated glass cover slips for 20 minutes and post-fixed at room temperature for 1 h in a solution containing 1% OsO4 in 0.1 M cacodylate buffer, pH 7.2. The material was then washed, dehydrated in an ethanol series (15%, 30%, 50%, 70%, 90% and 100%), critical point-dried in CO2 and mounted on specimen stubs sputtered with a thin layer of gold and observed in a Zeiss LEO 1530 scanning electron microscope.

### Transmission electron microscopy

Gametocytes were fixed in 2% glutaraldehyde/ 4% paraformaldehyde in 0.1 M phosphate buffer, pH 7.2. The material was washed in 0.1 M phosphate buffer, and post-fixed at room temperature in a solution containing 1% OsO4, 0.8% potassium ferrocyanide and 5mM CaCl_2_ in the same buffer. After 1h the material was washed in 0.1M phosphate buffer, dehydrated in an ascending acetone series (15%, 30%, 50%, 70%, 90% and 100%) and embedded in Polybed 812 resin. Sections were stained with uranyl acetate, followed by lead citrate, before observation in a FEI Tecnai Spirit 120kV microscope.

### Light microscopy

Imaging of live or fixed parasites was either performed on a Zeiss Axiovert 200 with a magnification of 25x/63x or on a Nikon spinning disc microscope using a 100x objective. Nuclei were visualised with Hoechst. For bloodstage parasites, one drop of infected tail blood was added to a microscope slide and covered with a cover glass. Image processing was done with ImageJ.

### Sporozoite motility

Gliding assays of isolated sporozoites were done in 96-well optical bottom plates (Nunc) using 3% BSA/ RPMI with a frame rate of 3 s for 4 min. Speed was determined with the manual tracking tool in ImageJ. For the detection of trails, sporozoites were activated to glide for 20 min and immediately fixed with 3% PFA in PBS.

### Secretion assay of sporozoites

Salivary gland sporozoites were isolated in PBS, grounded with a pestle and released sporozoites washed once with PBS and activated for 30 min at RT with 30 μL RPMI containing 0.5% Pluronic acid. Pellet and supernatant were separated by centrifugation and the pellet resuspended in 30 μL of RIPA buffer containing protease inhibitors. Samples were stored at -20°C until analyses by Western blot.

### Immunofluorescence staining of sporozoites

After fixation, cells were permeabilized with 0.5% TritonX for 15 minutes. Primary antibodies were incubated for 1hour and washed twice with PBS. Secondary antibodies were incubated for 1 hour washed twice with PBS and cells resuspended in PBS containing Hoechst for observation under the microscope. Images were either taken on a Zeiss 200M Axiovert widefield (63x) or Nikon spinning disc (100x) microscope. Image processing was performed with ImageJ. Rabbit anti Trap: 1/100 (P. Sinnis), rabbit anti GFP for IFA 1/200 (abfinity), mouse anti GFP for western blot: 1/1000 (4mg/ml Roche). Goat anti mouse 594 1/1000 (Invitrogen 2mg/ml), goat anti rabbit 594 1/1000 (Invitrogen 2mg/ml), goat anti mouse HRP 1/10000 (GE Healthcare).

### Mosquito infection

In a standard feeding assay 100 female *Anopheles stephensi* mosquitoes were allowed to feed for 20 minutes on two infected and anaesthetized mice [Ketamin/ Xylazin (2.5mg/0.25mg)] 3 days after transfer of 20,000,000 infected RBC into a naïve mouse. Post-infection mosquitoes were kept in an incubator set to 21°C. Midgut sporozoites were counted at days [10;14] and SGS were counted at days [17;25]. Oocysts were counted between day 10 and 15 either after staining of isolated midguts with 0.1% mercurochrome in PBS.

## Supporting Information

S1 Fig List of *Plasmodium berghei* and *P*. *falciparum* major facilitator superfamily transporters. SP signal peptide; TM number of transmembrane domains.
(TIF)

S2 Fig ClustalW alignment of *Plasmodium berghei* PAT with proteins from related *Plasmodium* species. PKH *Plasmodium knowlesi*; PVX *P*. *vivax*; PF3D7 *P*. *falciparum*; PBANKA *P*. *berghei*; PYYM *P*. *yoelii*; PCHAS *P*. *chabaudi*.All sequences from www.plasmodb.org. Shading as provided by boxshade (www.ch.embnet.org).
(TIF)

S3 Fig ClustalW alignment of *Plasmodium berghei* PAT with homologous proteins from selected apicomplexans. NCLIV *Neospora caninum*; TGGT1 *Toxoplasma gondii*; ETH *Eimeria tenella*. All sequences from www.plasmodb.org or www.toxodb.org. Shading as provided by boxshade (www.ch.embnet.org).
(TIF)

S4 Fig Expression profiles determined by microarray of known *Plasmodium* invasion factors and genes that display similar trends and includes *pat*. Data from Gomes et al. 2011 [36].
(TIF)

S5 Fig Expression profiling of *pat* across the *P*. *berghei* life cycle by Reverse Transcriptase-PCR. (TIF)

S6 Fig Genotyping of *pat*::*gfp parasite* clone. (A) Agarose gels of PCRs using wildtype and mutant parasite DNA. (B) Reactions as indicated on the top right. (C) Genomic loci and position of primers.
(TIF)

S7 Fig Genotyping of Δ*pat* parasite clone. (A) Agarose gels of PCRs using wildtype and mutant parasite DNA. (B) Reactions as indicated on the top right. (C) Genomic loci and position of primers.
(TIF)

S8 Fig Genotyping of *P*. *berghei pat* ^*PBANKA*^::*mcherry* in Δ*pat* parasite clone. (A) Agarose gels of PCRs using wildtype and mutant parasite DNA. (B) Reactions as indicated on the top right. (C) Genomic loci and position of primers.
(TIF)

S9 Fig Genotyping of *P*. *falciparum pat* ^*PF3D7*^::*mcherry* in Δ*pat* parasite clone. (A) Agarose gels of PCRs using wildtype and mutant parasite DNA. (B) Reactions as indicated on the top right. (C) Genomic loci and position of primers.
(TIF)

S10 Fig Genotyping of *g377*::*mcherry* in *pat*::*gfp parasite* clone. (A) Agarose gels of PCRs using wildtype and mutant parasite DNA. (B) Reactions as indicated on the top right. (C) Genomic loci and position of primers.
(TIF)

S11 Fig Genotyping of *pplp2*::*mcherry* in *pat*::*gfp parasite* clone. (A) Agarose gels of PCRs using wildtype and mutant parasite DNA. (B) Reactions as indicated on the top right. (C) Genomic loci and position of primers.
(TIF)

S12 Fig Genotyping of *g377*::*mcherry* in Δ*pat* parasite clone. (A) Agarose gels of PCRs using wildtype and mutant parasite DNA. (B) Reactions as indicated on the top right. (C) Genomic loci and position of primers.
(TIF)

S13 Fig Genotyping of *pplp2*::*mcherry* in Δ*pat* parasite clone. (A) Agarose gels of PCRs using wildtype and mutant parasite DNA. (B) Reactions as indicated on the top right. (C) Genomic loci and position of primers.
(TIF)

S14 Fig Time course localization of G377::mCherry in the *pat*::*gfp*background during gamete egress. Initially distributed within the cytoplasm, G377+ and PAT+ vesicles traffic to the plasma membrane during activation. Scale bar = 5 μm.
(TIF)

S15 Fig Time course of localization G377::mCherry in Δ*pat* background during gamete egress. Initially distributed within the cytoplasm, G377+ vesicles traffic to the plasma membrane during activation, but fail to lyse the the red blood cell membrane. Scale bar = 5 μm.
(TIF)

S16 Fig Genotyping of *ss*::*gfp*::*trap parasite* clone. (A) Protein motifs of wildtype TRAP and SS::GFP::TRAP. TSR thrombospondin repeat; JMD juxtamembrane region; TM transmembrane domain. (B) Agarose gels of PCRs using wildtype and mutant parasite DNA. (C) Reactions as indicated on the top right. (D) Genomic loci and position of primers.
(TIF)

S17 Fig Genotyping of promoter swap mutant clone *pat* ^*ccp*.*PP*^. (A) Agarose gels of PCRs using wildtype and mutant parasite DNA. (B) Reactions as indicated on the top right. (C) Genomic loci and position of primers.
(TIF)

S18 Fig Genotyping of *ss*::*gfp*::*trap in the pat* ^*ccp*.*PP*^ *background*. (A) Agarose gels of PCRs using wildtype and mutant parasite DNA. (B) Reactions as indicated on the top right. (C) Genomic loci and position of primers.
(TIF)

S19 Fig ClustalW alignment of yeast (*Saccharomyces cerevisiae*) FEN2 (www.yeastgenome.org) and *P*. *berghei* (PBANKA) PAT. Transmembrane domains as predicted by TMHHM @ cbs.dtu.dk: black +: *P*. *berghei* transmembrane domains; red +: two additional *S*. *cerevisiae* transmembrane domains. Shading as provided by boxshade (www.ch.embnet.org).
(TIF)

S20 Fig ClustalW alignment *P*. *berghei* (PBANKA) PAT and fly (*Drosophila melanogaster*) Spinster (ACD81856.1; www.ncbi.nlm.nih.gov). Transmembrane domains as predicted by TMHHM @ cbs.dtu.dk: black +: *P*. *berghei* transmembrane domains; red +: two additional *D*. *melanogaster* transmembrane domains. Shading as provided by boxshade (www.ch.embnet.org).
(TIF)

S21 Fig ClustalW alignment *P*. *berghei* (PBANKA_030390) PAT and *Arabidopsis thaliana* XP_010437304.1 (www.ncbi.nlm.nih.gov). Shading as provided by boxshade (www.ch.embnet.org).
(TIF)

S1 Movie Egress (exflagellation) of wildtype male gametes. (AVI)

S2 Movie Failed egress (exflagellation) of *pat* male gametes. Trapped within the erythrocyte, gametes are still motile.
(AVI)
