# Supplementary figures and images for: A Putative Small Solute Transporter Is Responsible for the Secretion of G377 and TRAP-Containing Secretory Vesicles during Plasmodium Gamete Egress and Sporozoite Motility
Source: PLoS Pathog. 2016 Jul 18;12(7):e1005734. doi: 10.1371/journal.ppat.1005734 (PMC4948853; doi:10.1371/journal.ppat.1005734)

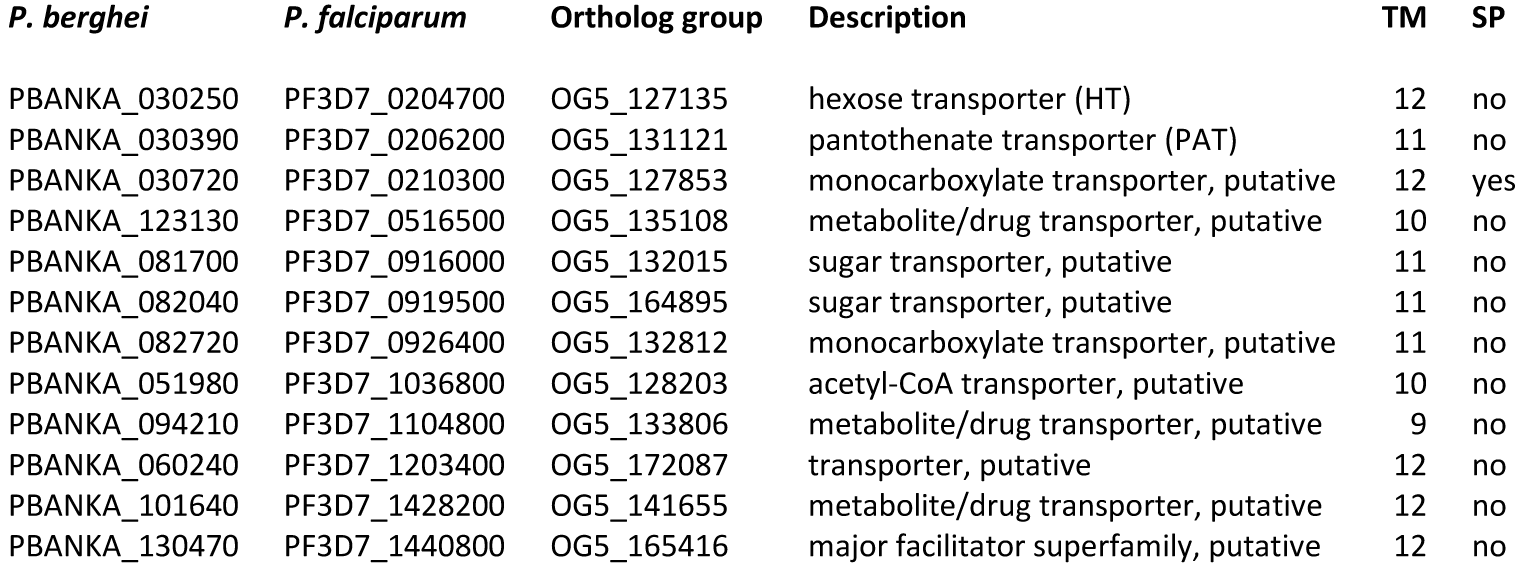

Supplement: S1 Fig — SP signal peptide; TM number of transmembrane domains. (TIF) [file ppat.1005734.s001.tif]

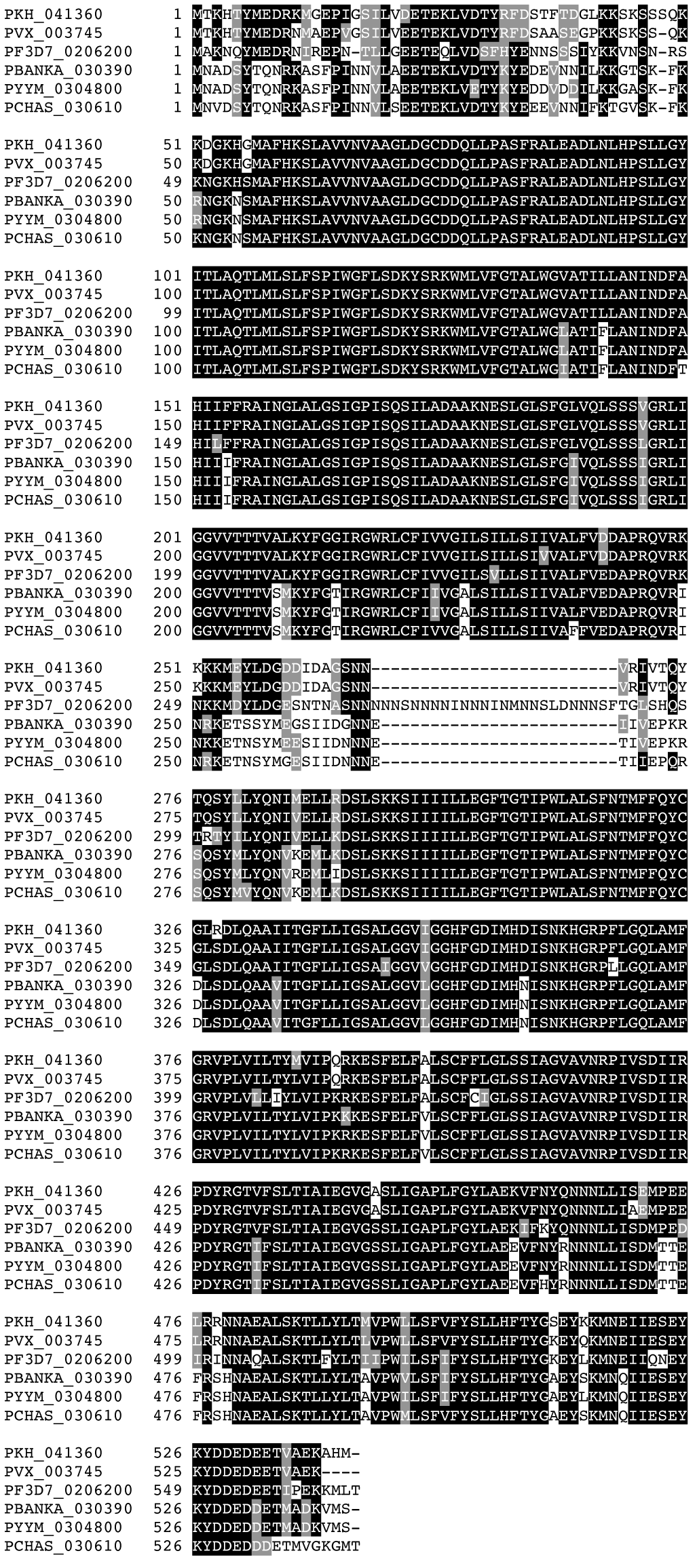

Supplement: S2 Fig — PKH Plasmodium knowlesi; PVX P. vivax; PF3D7 P. falciparum; PBANKA P. berghei; PYYM P. yoelii; PCHAS P. chabaudi.All sequences from www.plasmodb.org. Shading as provided by boxshade (www.ch.embnet.org). (TIF) [file ppat.1005734.s002.tif]

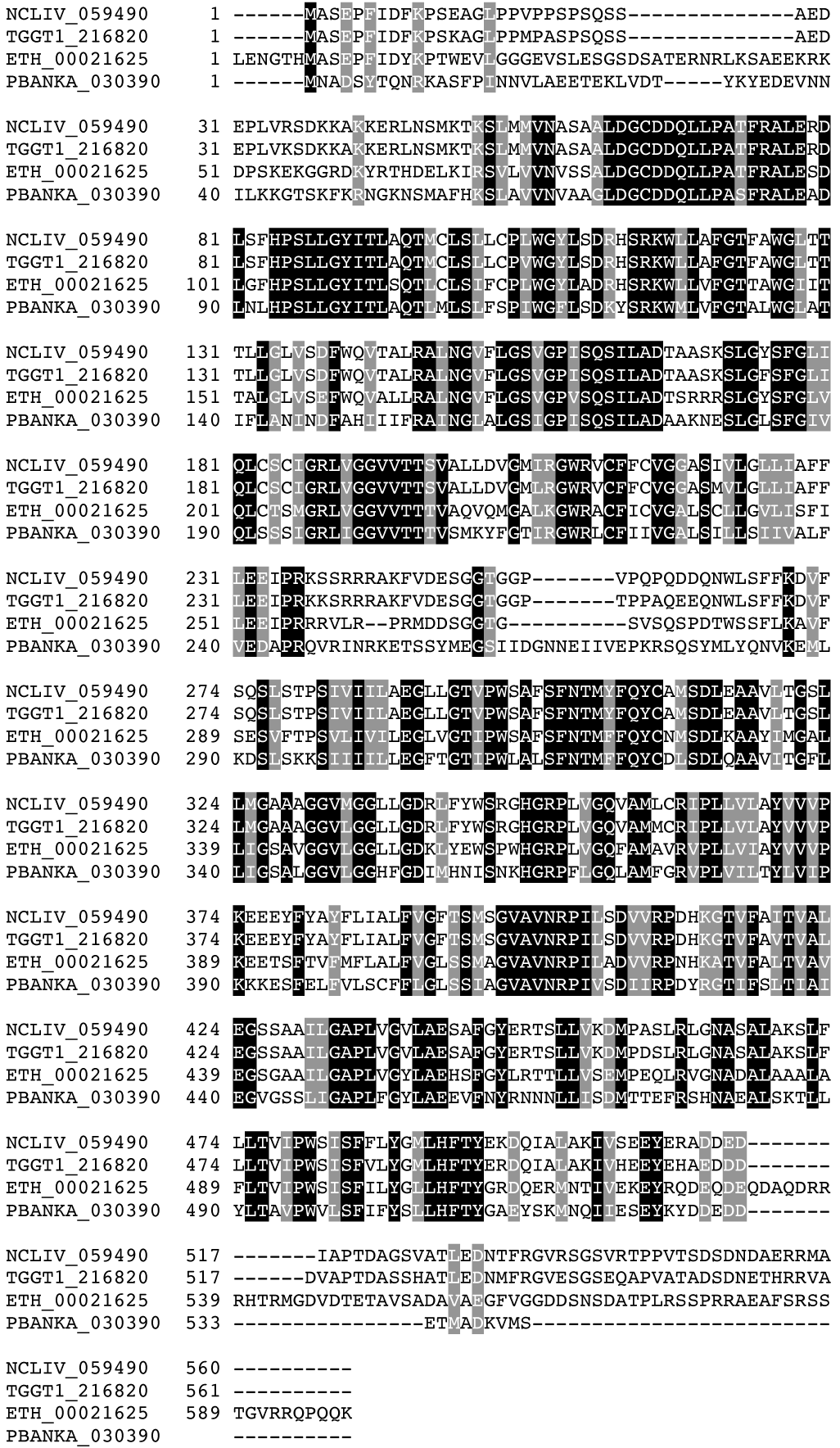

Supplement: S3 Fig — NCLIV Neospora caninum; TGGT1 Toxoplasma gondii; ETH Eimeria tenella. All sequences from www.plasmodb.org or www.toxodb.org. Shading as provided by boxshade (www.ch.embnet.org). (TIF) [file ppat.1005734.s003.tif]

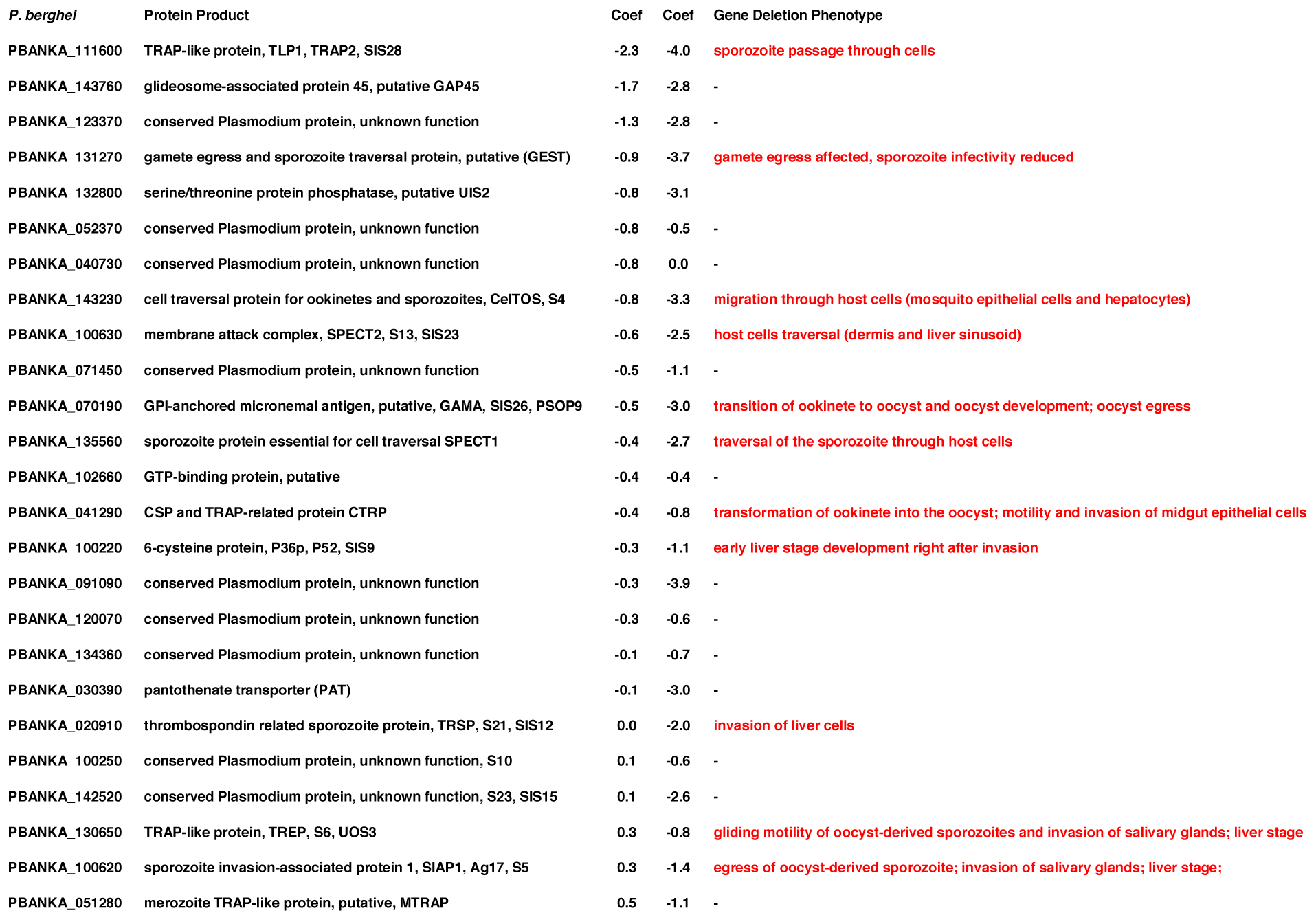

Supplement: S4 Fig — Data from Gomes et al. 2011 [36]. (TIF) [file ppat.1005734.s004.tif]

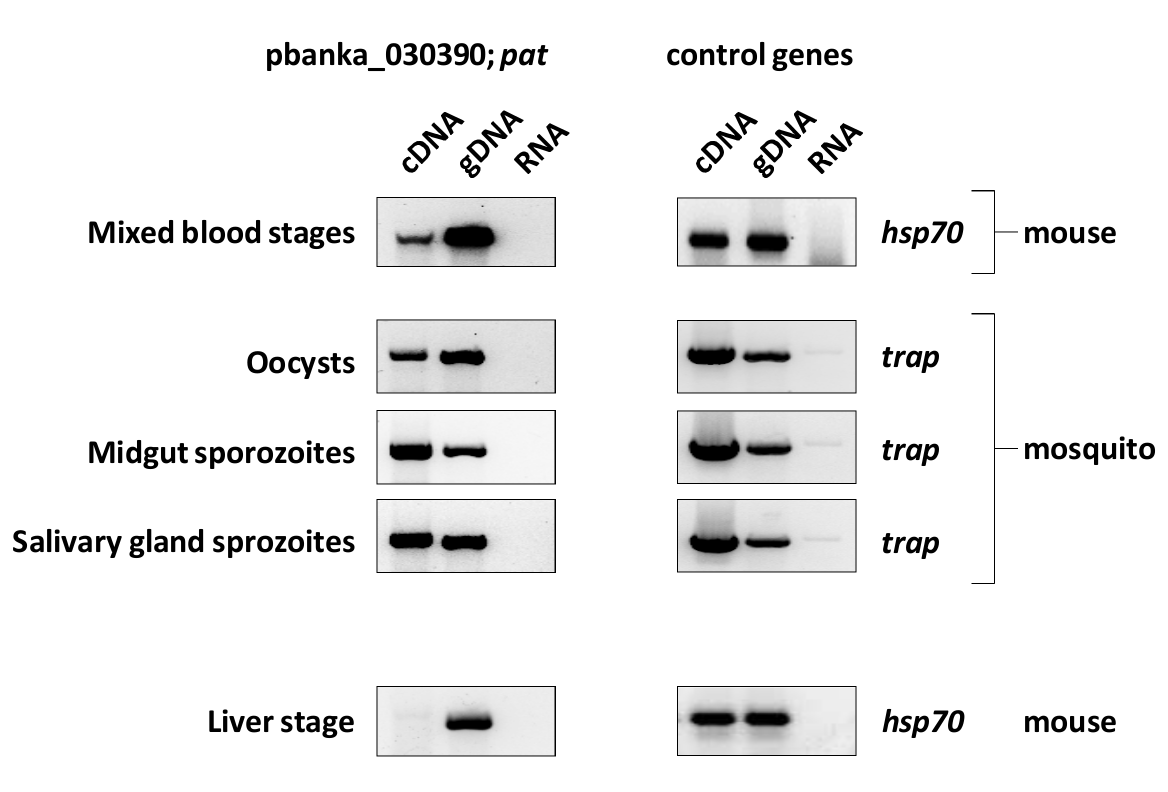

Supplement: S5 Fig — (TIF) [file ppat.1005734.s005.tif]

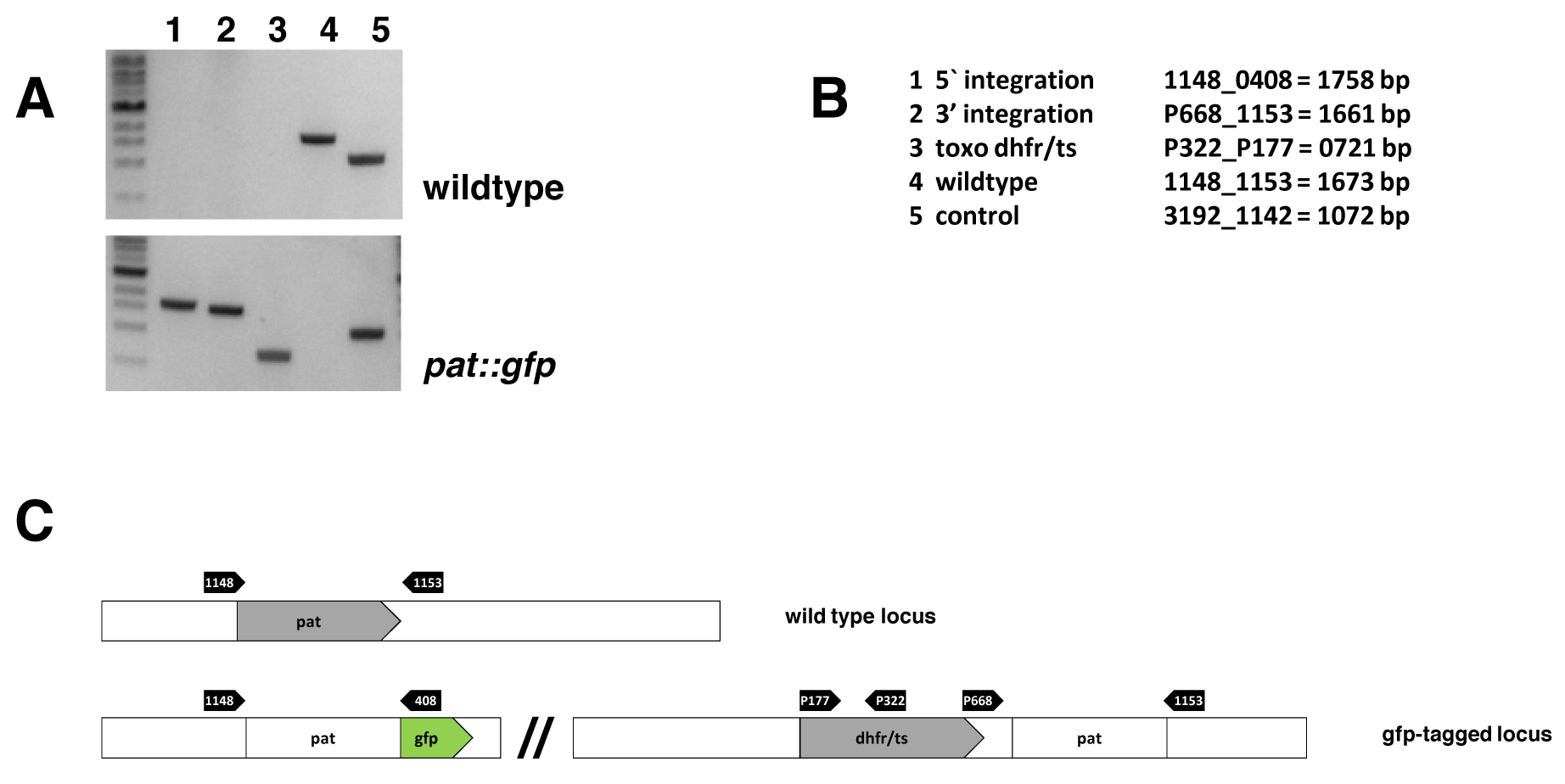

Supplement: S6 Fig — (A) Agarose gels of PCRs using wildtype and mutant parasite DNA. (B) Reactions as indicated on the top right. (C) Genomic loci and position of primers. (TIF) [file ppat.1005734.s006.tif]

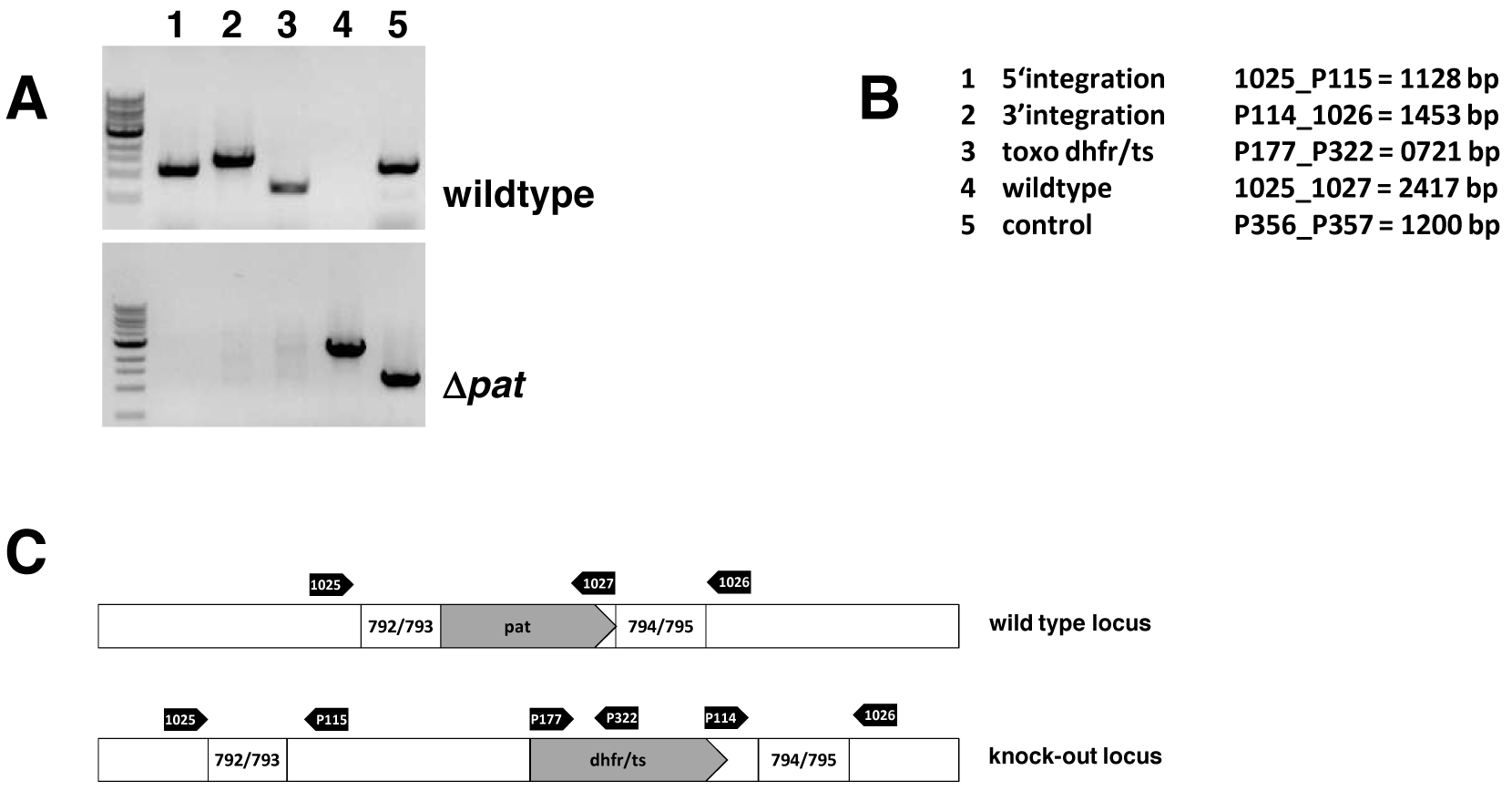

Supplement: S7 Fig — (A) Agarose gels of PCRs using wildtype and mutant parasite DNA. (B) Reactions as indicated on the top right. (C) Genomic loci and position of primers. (TIF) [file ppat.1005734.s007.tif]

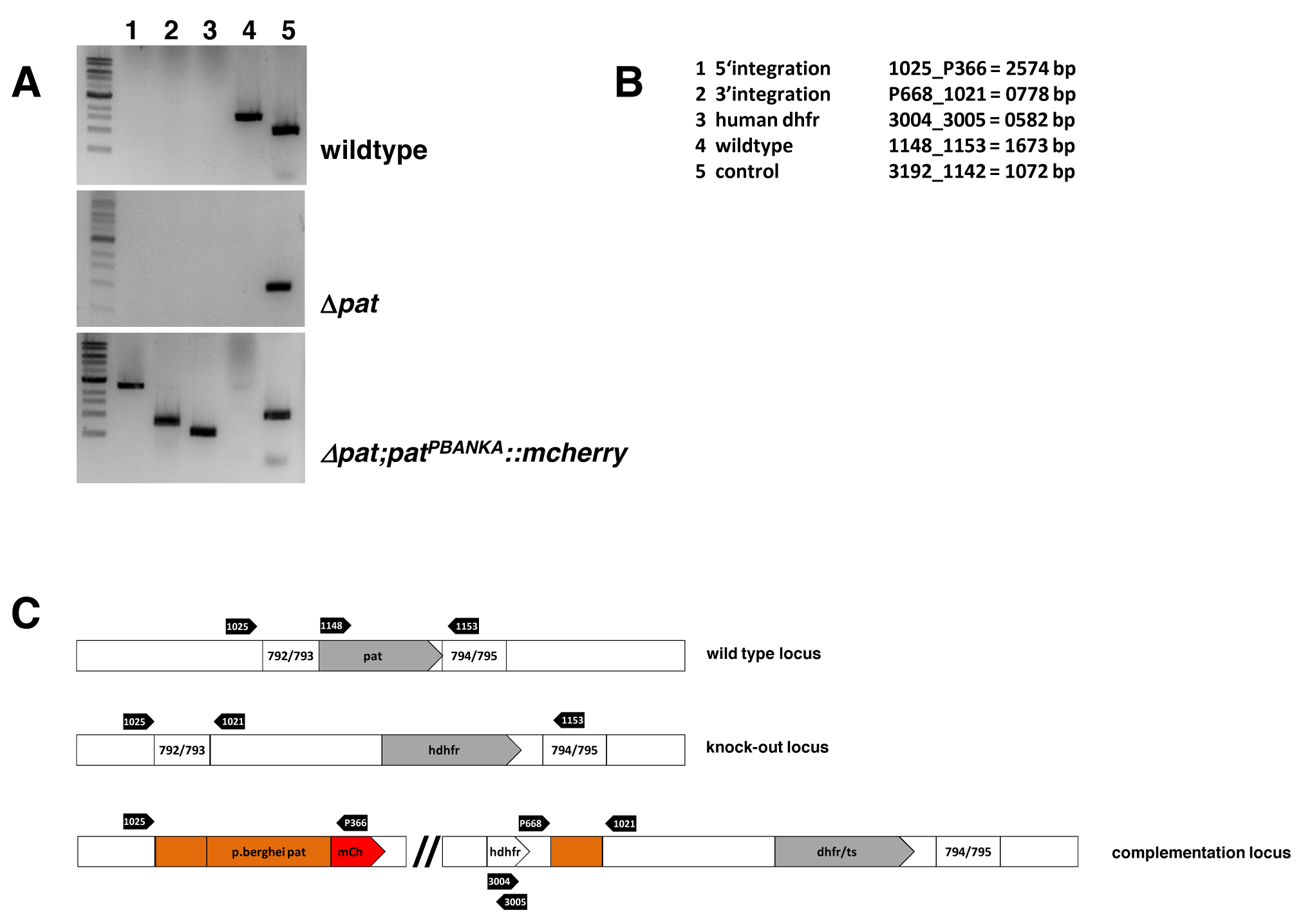

Supplement: S8 Fig — (A) Agarose gels of PCRs using wildtype and mutant parasite DNA. (B) Reactions as indicated on the top right. (C) Genomic loci and position of primers. (TIF) [file ppat.1005734.s008.tif]

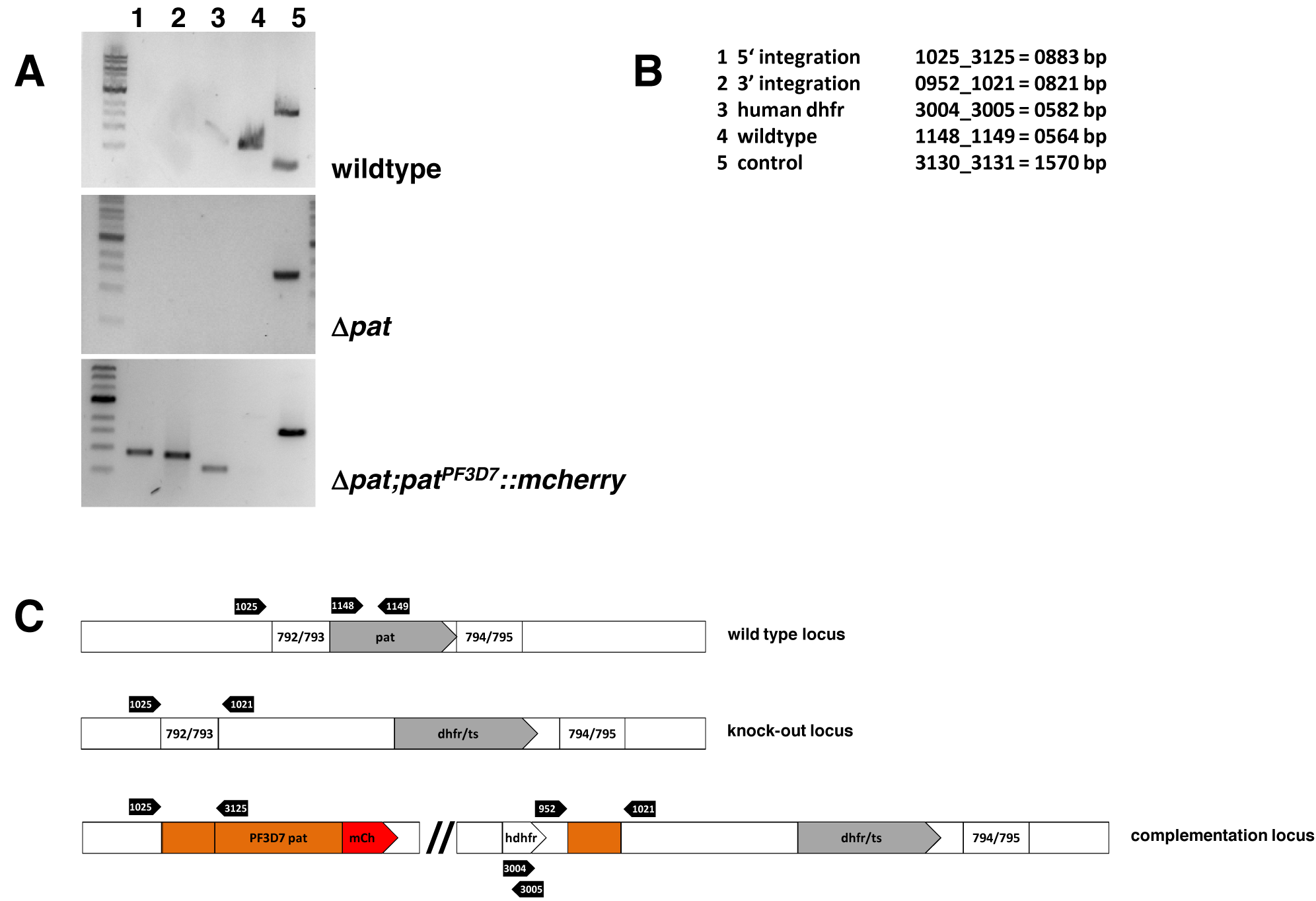

Supplement: S9 Fig — (A) Agarose gels of PCRs using wildtype and mutant parasite DNA. (B) Reactions as indicated on the top right. (C) Genomic loci and position of primers. (TIF) [file ppat.1005734.s009.tif]

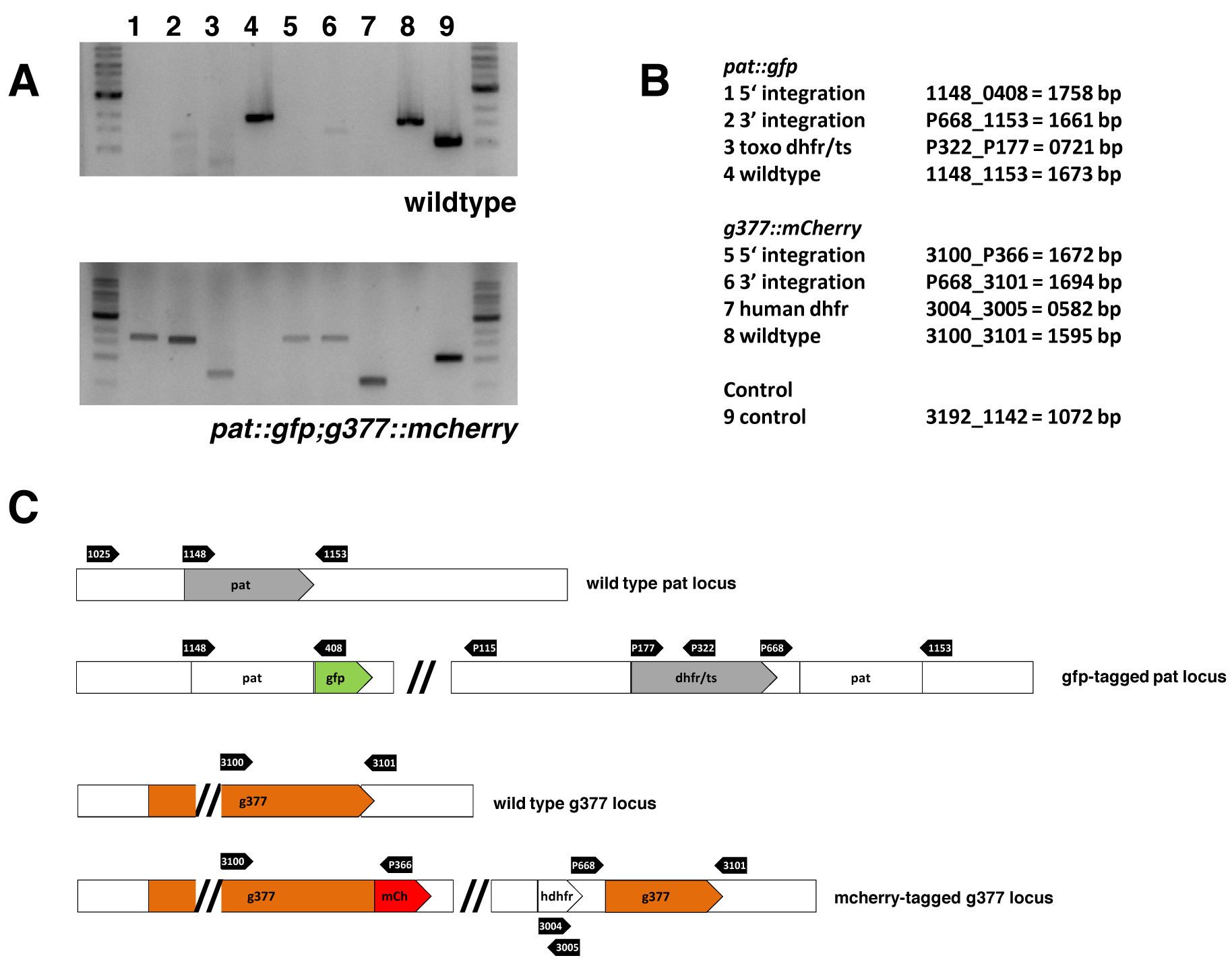

Supplement: S10 Fig — (A) Agarose gels of PCRs using wildtype and mutant parasite DNA. (B) Reactions as indicated on the top right. (C) Genomic loci and position of primers. (TIF) [file ppat.1005734.s010.tif]

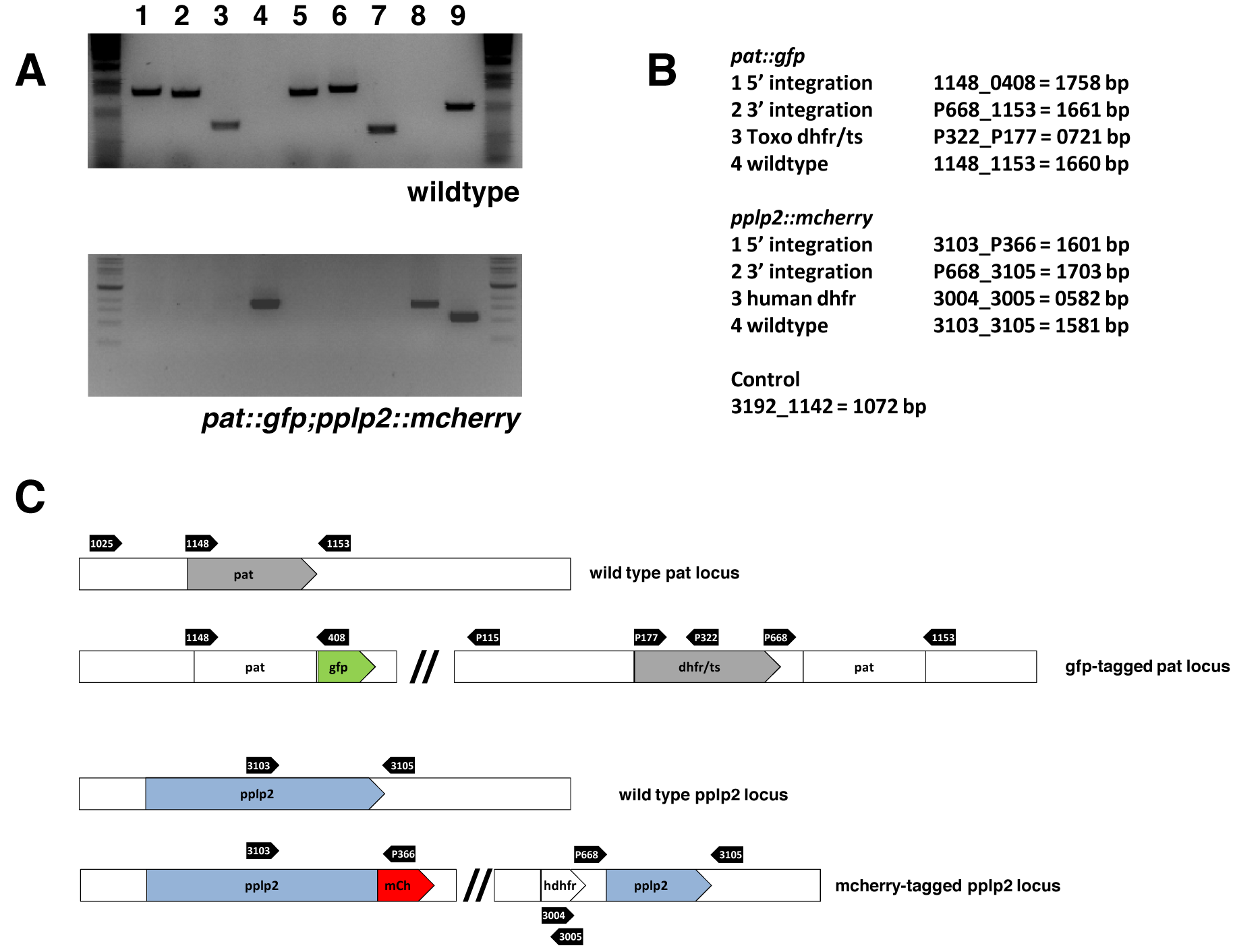

Supplement: S11 Fig — (A) Agarose gels of PCRs using wildtype and mutant parasite DNA. (B) Reactions as indicated on the top right. (C) Genomic loci and position of primers. (TIF) [file ppat.1005734.s011.tif]

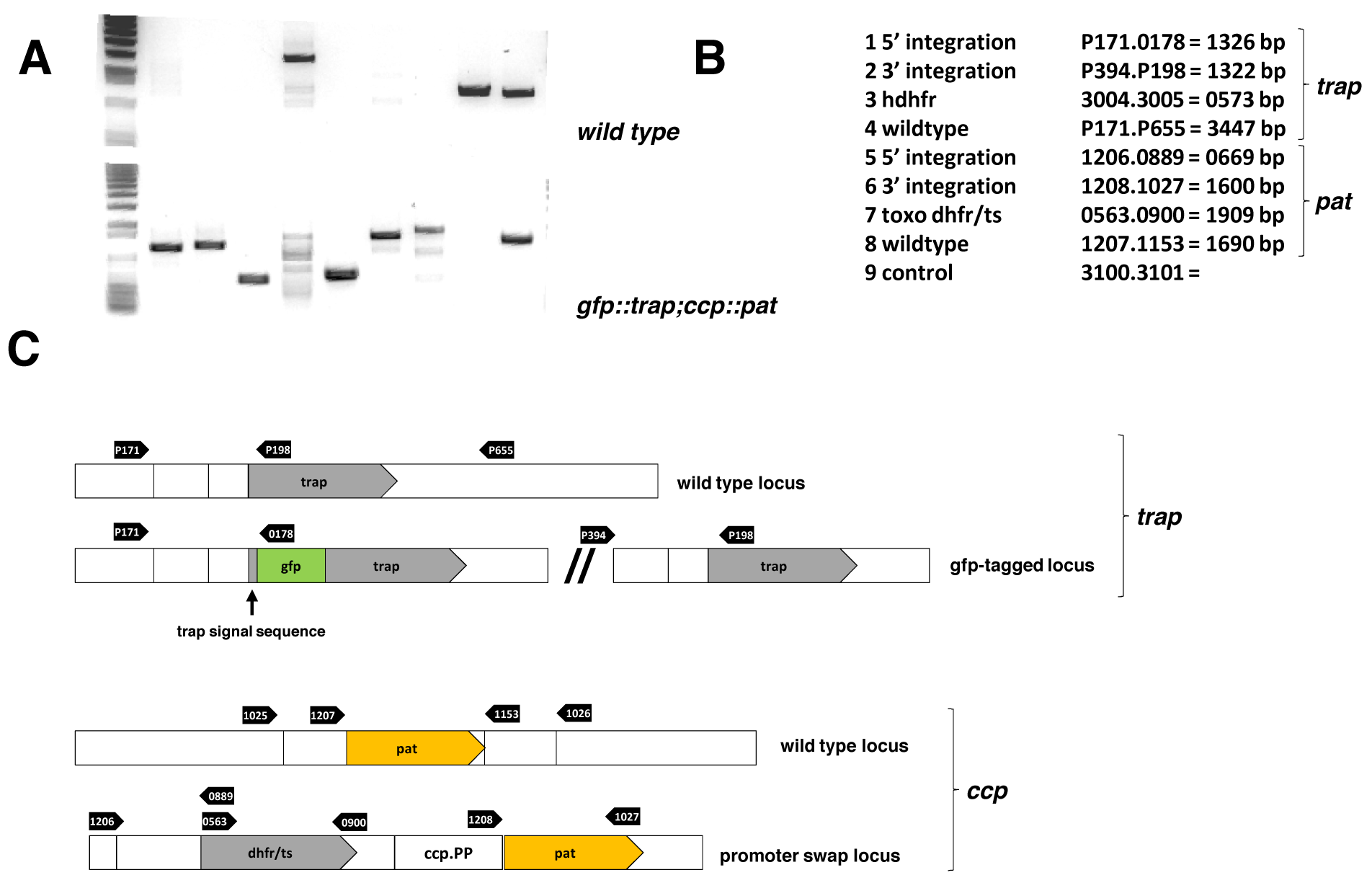

Supplement: S12 Fig — (A) Agarose gels of PCRs using wildtype and mutant parasite DNA. (B) Reactions as indicated on the top right. (C) Genomic loci and position of primers. (TIF) [file ppat.1005734.s012.tif]

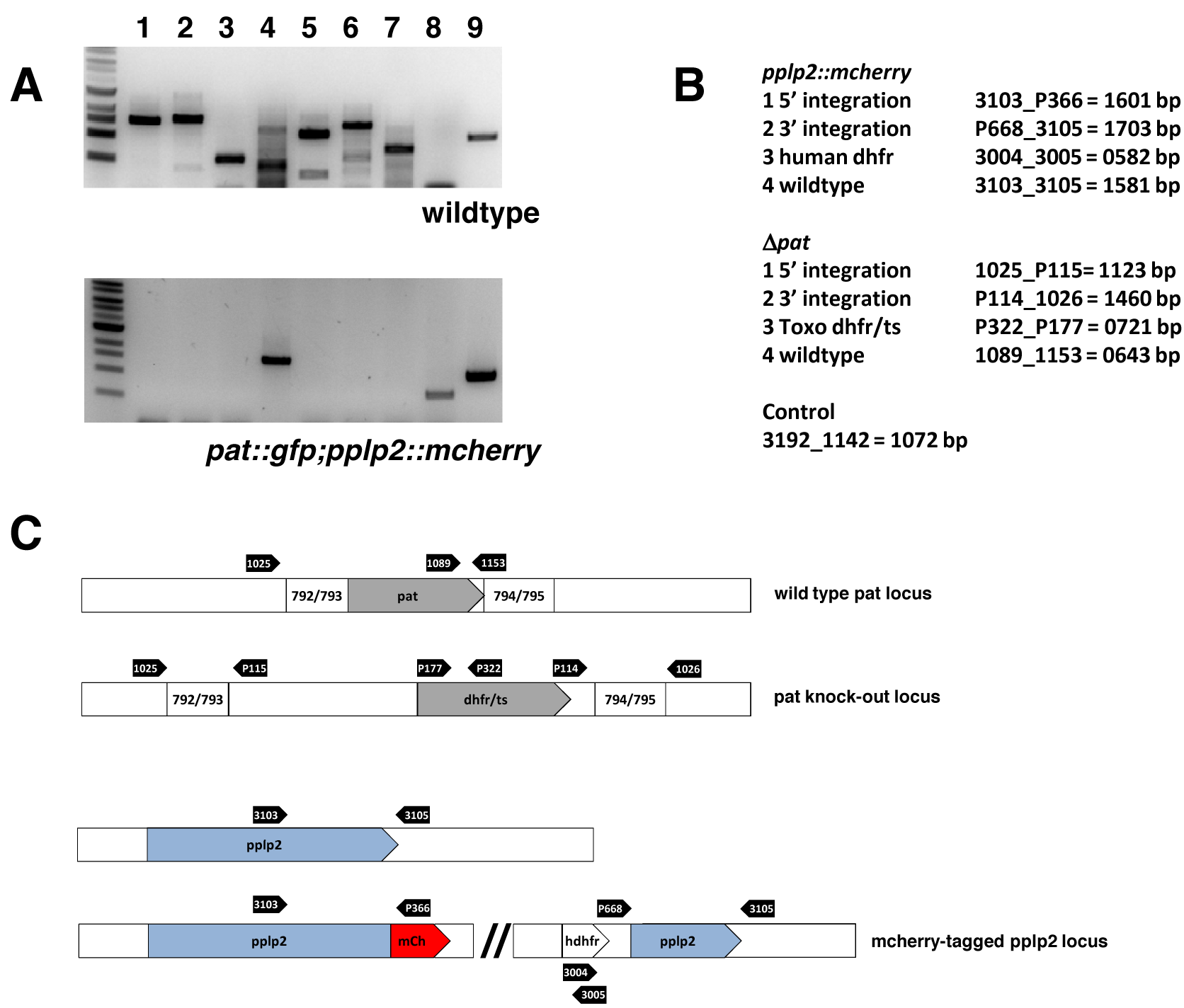

Supplement: S13 Fig — (A) Agarose gels of PCRs using wildtype and mutant parasite DNA. (B) Reactions as indicated on the top right. (C) Genomic loci and position of primers. (TIF) [file ppat.1005734.s013.tif]

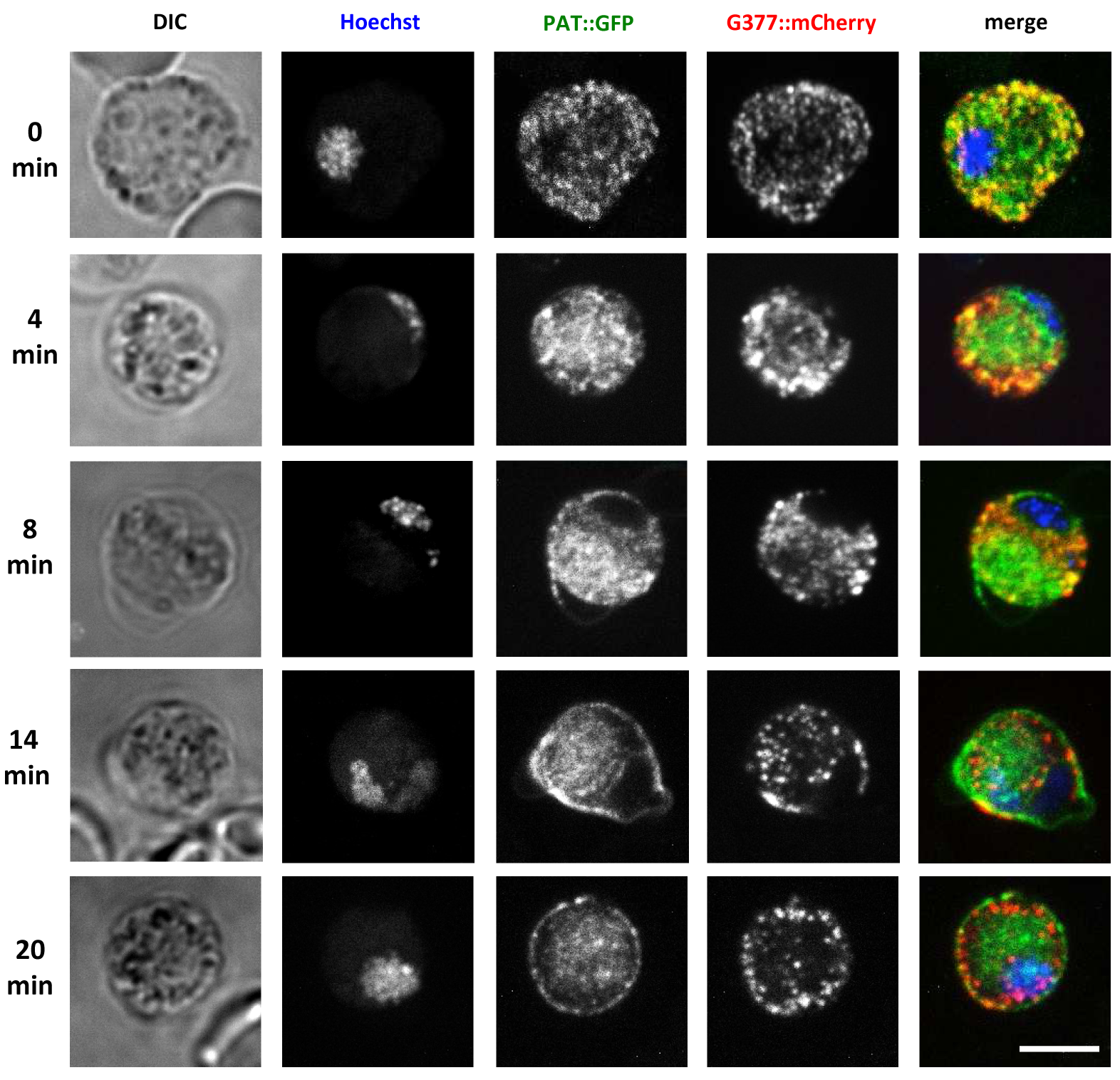

Supplement: S14 Fig — Initially distributed within the cytoplasm, G377+ and PAT+ vesicles traffic to the plasma membrane during activation. Scale bar = 5 μm. (TIF) [file ppat.1005734.s014.tif]

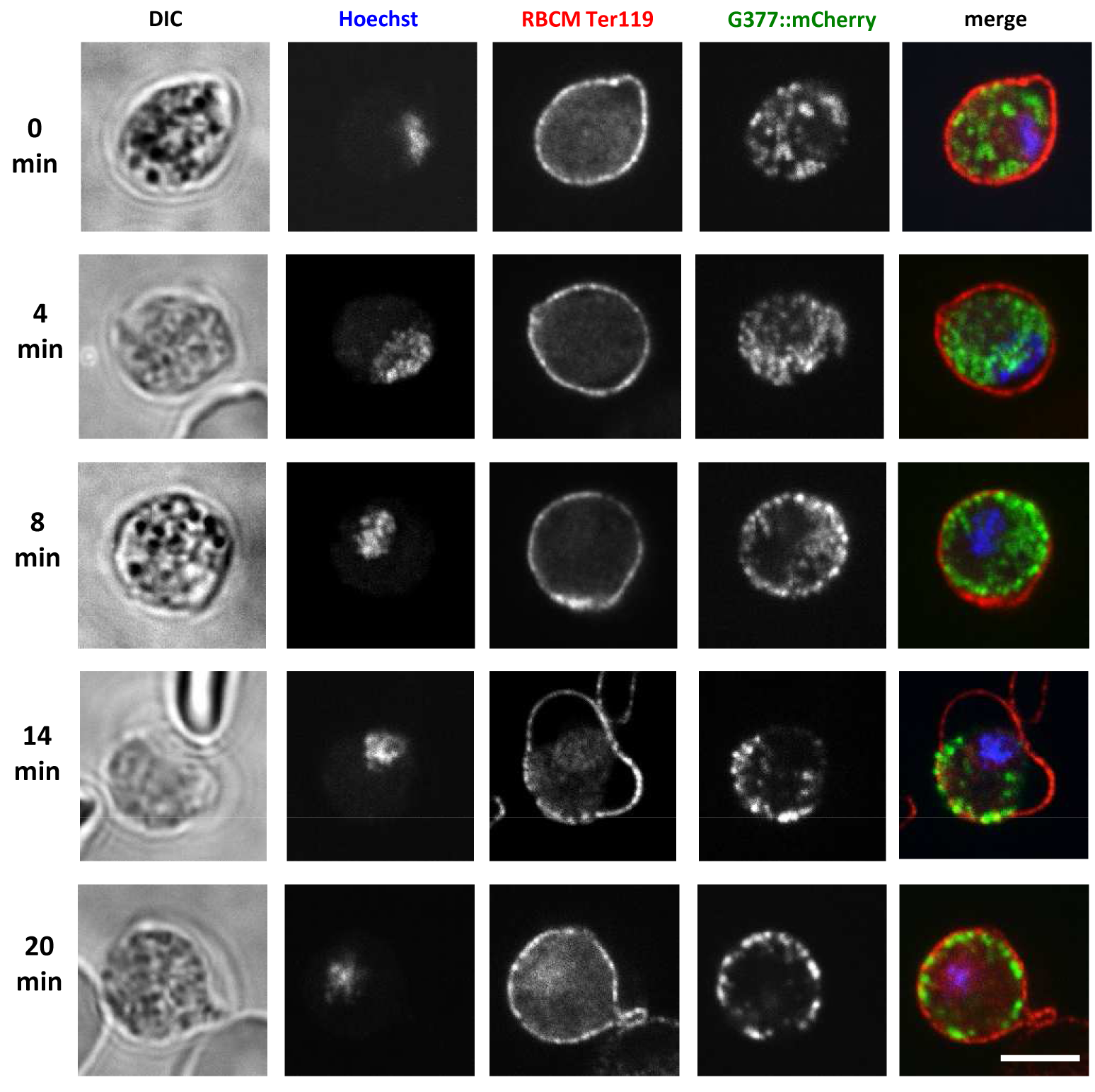

Supplement: S15 Fig — Initially distributed within the cytoplasm, G377+ vesicles traffic to the plasma membrane during activation, but fail to lyse the the red blood cell membrane. Scale bar = 5 μm. (TIF) [file ppat.1005734.s015.tif]

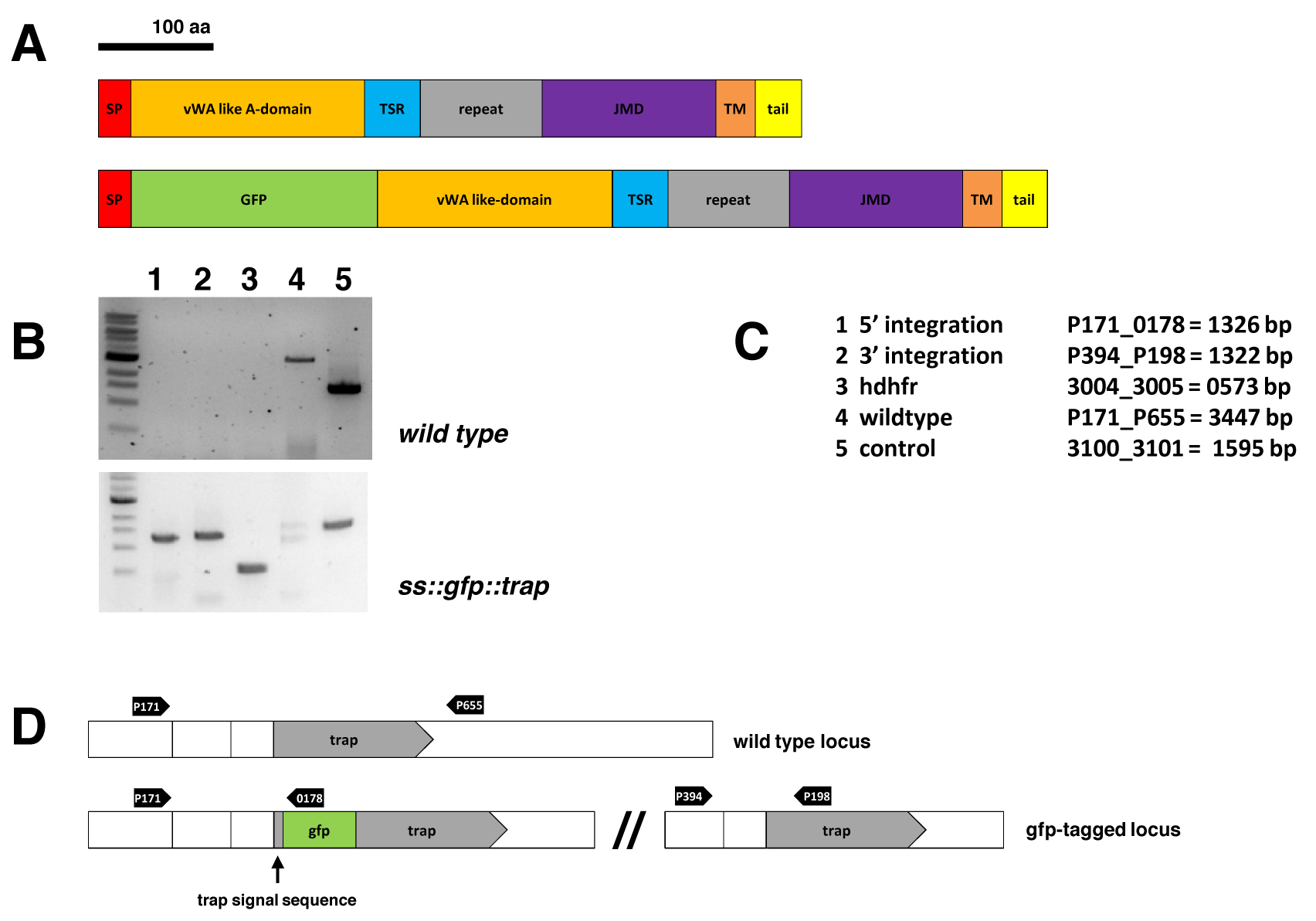

Supplement: S16 Fig — (A) Protein motifs of wildtype TRAP and SS::GFP::TRAP. TSR thrombospondin repeat; JMD juxtamembrane region; TM transmembrane domain. (B) Agarose gels of PCRs using wildtype and mutant parasite DNA. (C) Reactions as indicated on the top right. (D) Genomic loci and position of primers. (TIF) [file ppat.1005734.s016.tif]

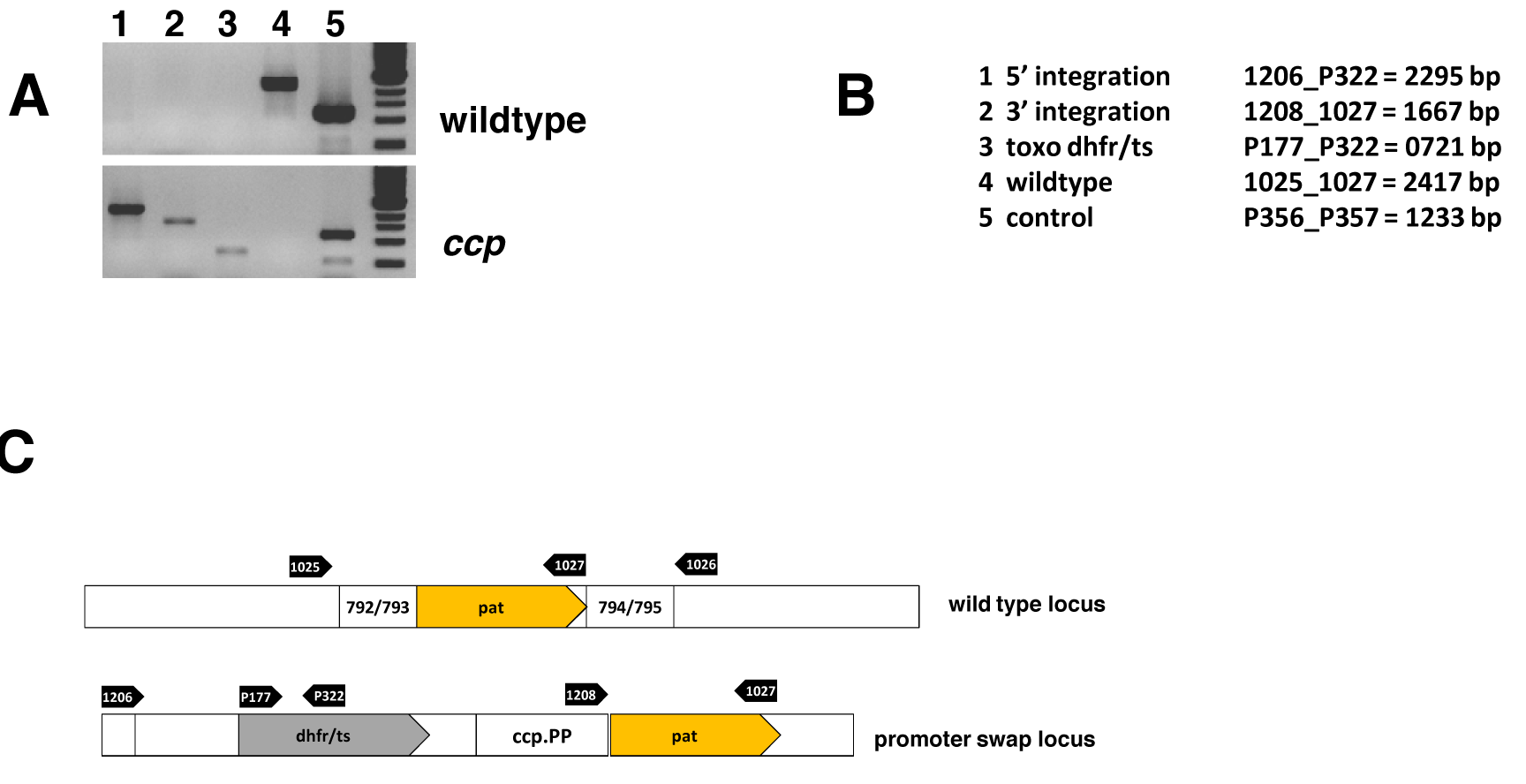

Supplement: S17 Fig — (A) Agarose gels of PCRs using wildtype and mutant parasite DNA. (B) Reactions as indicated on the top right. (C) Genomic loci and position of primers. (TIF) [file ppat.1005734.s017.tif]

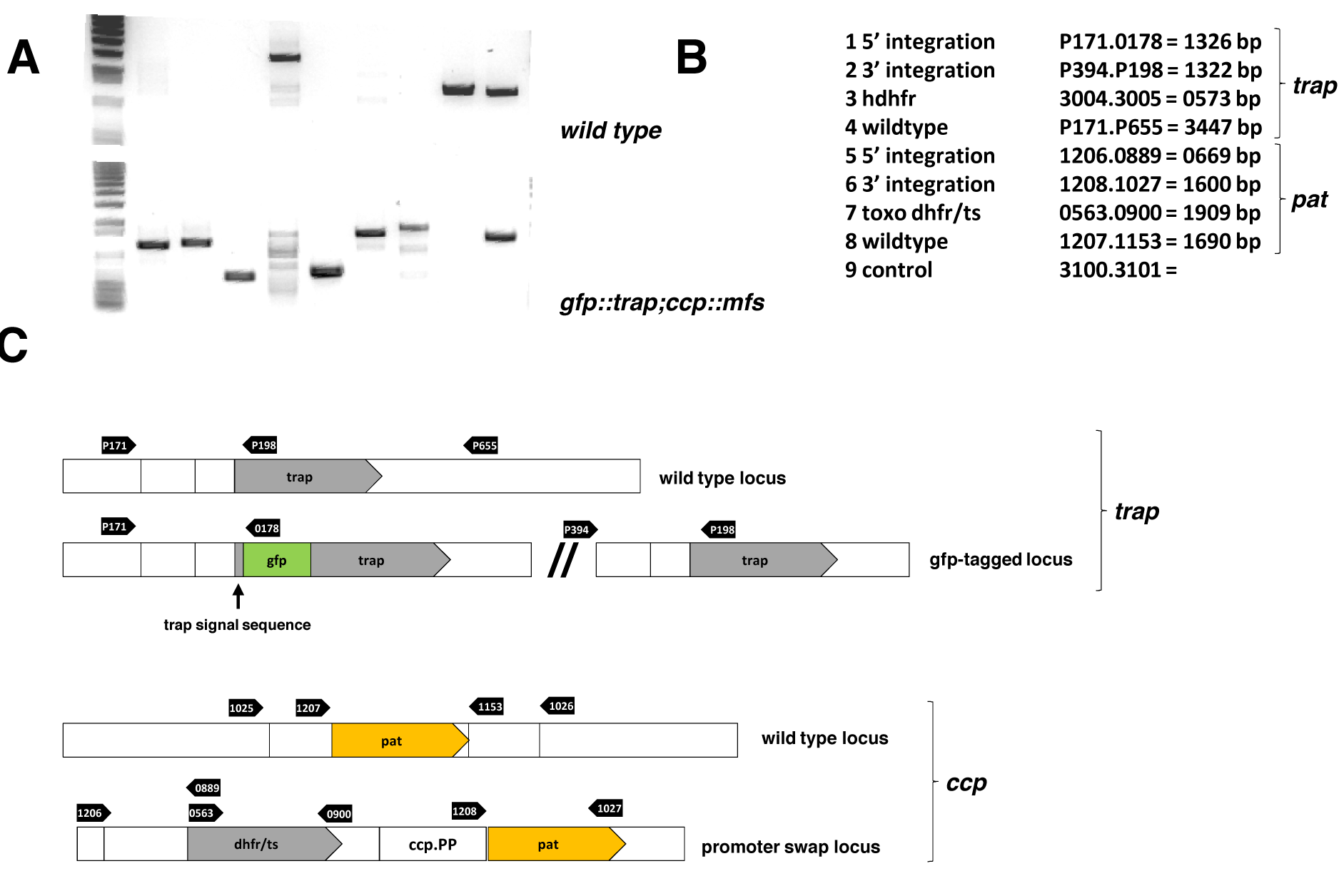

Supplement: S18 Fig — (A) Agarose gels of PCRs using wildtype and mutant parasite DNA. (B) Reactions as indicated on the top right. (C) Genomic loci and position of primers. (TIF) [file ppat.1005734.s018.tif]

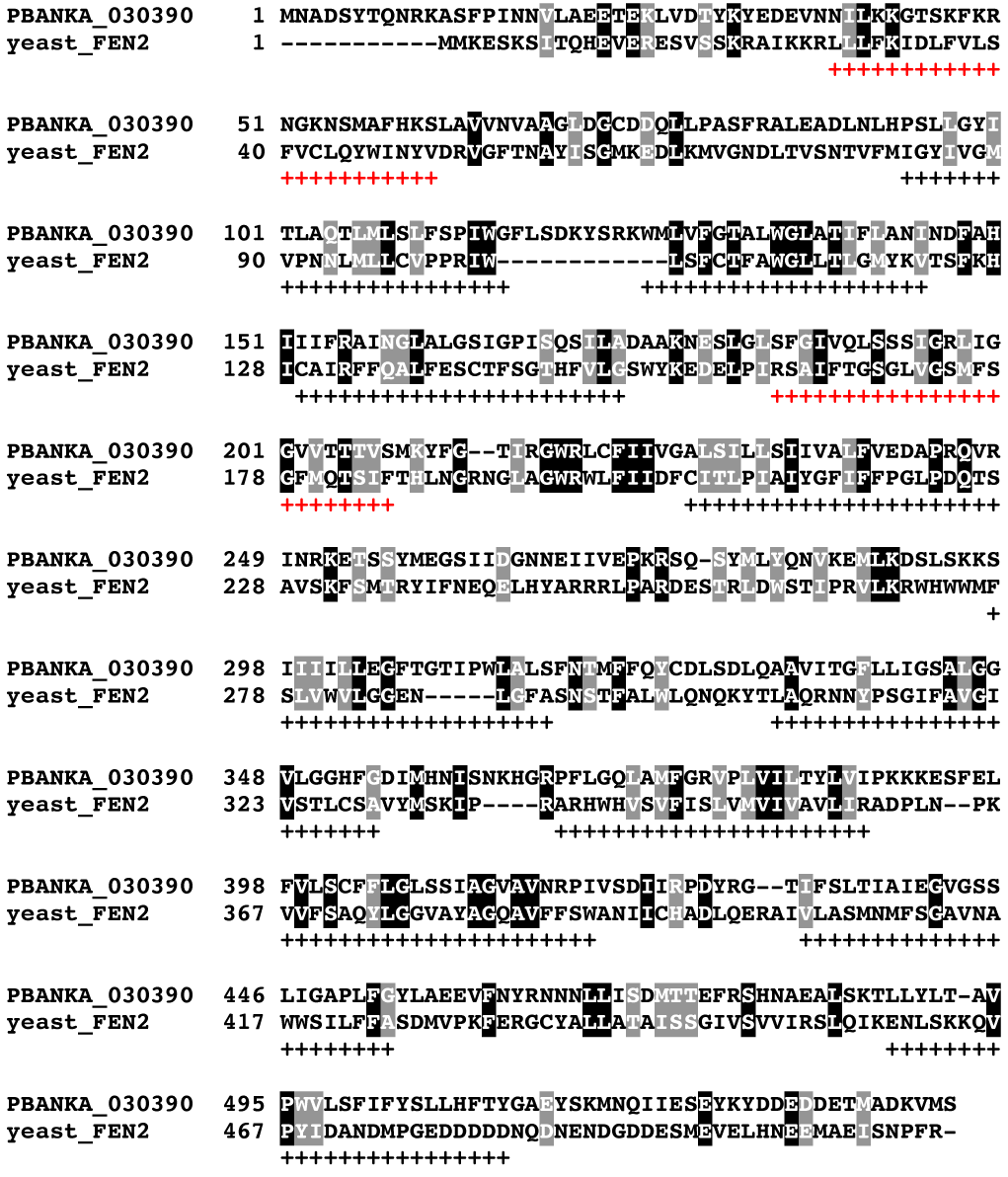

Supplement: S19 Fig — Transmembrane domains as predicted by TMHHM @ cbs.dtu.dk: black +: P. berghei transmembrane domains; red +: two additional S. cerevisiae transmembrane domains. Shading as provided by boxshade (www.ch.embnet.org). (TIF) [file ppat.1005734.s019.tif]

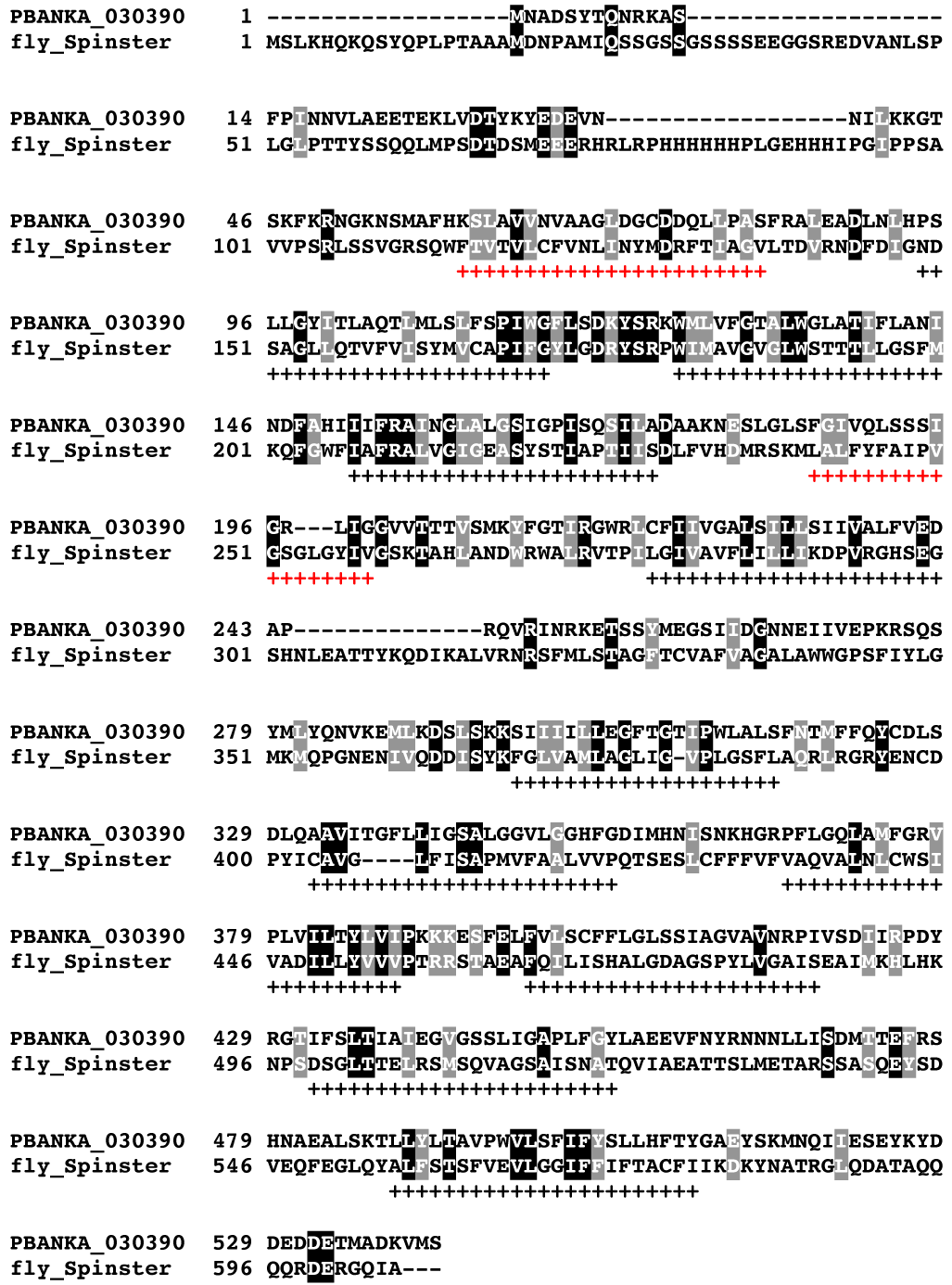

Supplement: S20 Fig — Transmembrane domains as predicted by TMHHM @ cbs.dtu.dk: black +: P. berghei transmembrane domains; red +: two additional D. melanogaster transmembrane domains. Shading as provided by boxshade (www.ch.embnet.org). (TIF) [file ppat.1005734.s020.tif]

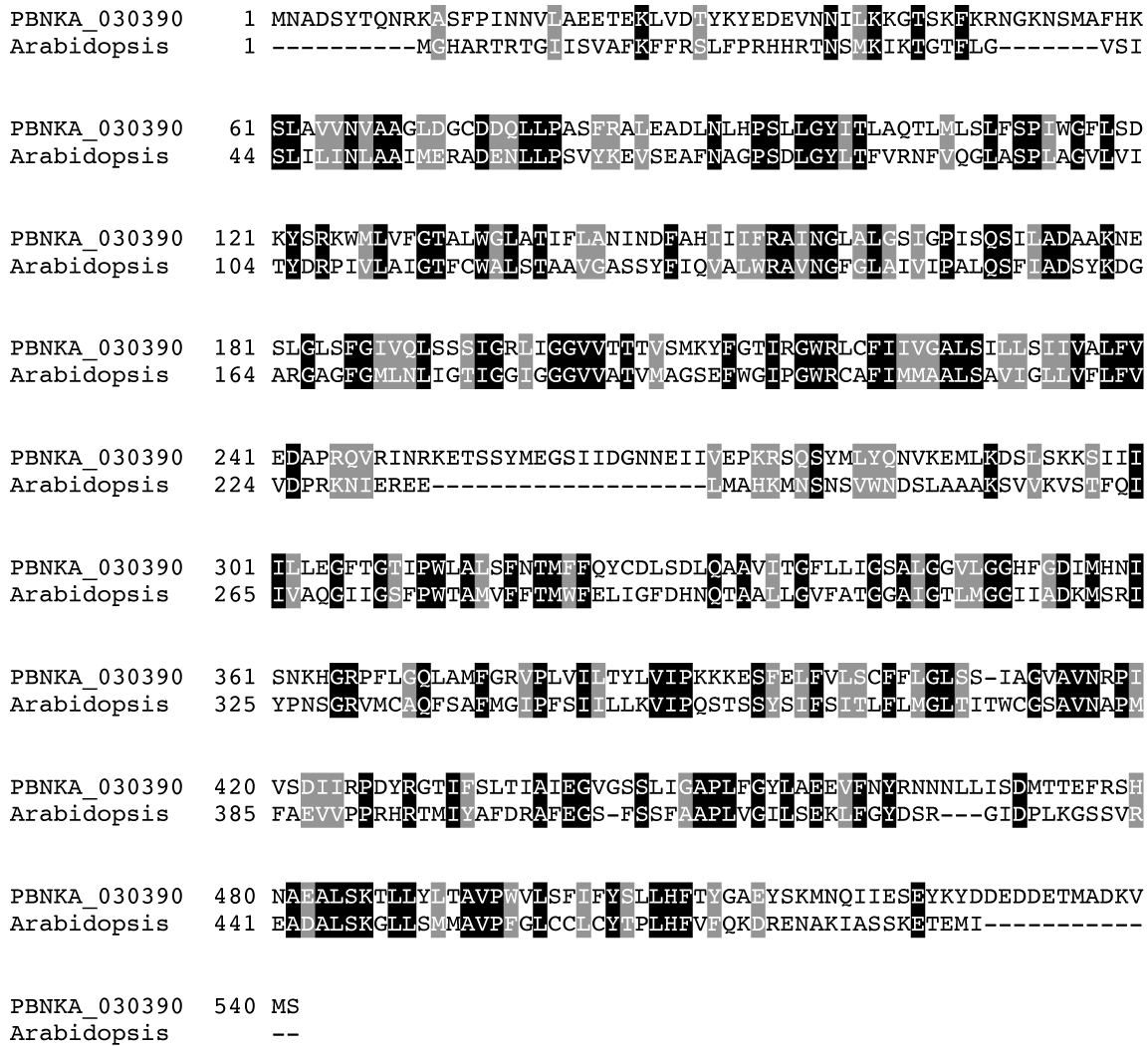

Supplement: S21 Fig — Shading as provided by boxshade (www.ch.embnet.org). (TIF) [file ppat.1005734.s021.tif]
